# The ‘*Eat Well @ IGA*’ healthy supermarket randomised controlled trial: process evaluation

**DOI:** 10.1186/s12966-021-01104-z

**Published:** 2021-03-12

**Authors:** Miranda R. Blake, Gary Sacks, Christina Zorbas, Josephine Marshall, Liliana Orellana, Amy K. Brown, Marj Moodie, Cliona Ni Mhurchu, Jaithri Ananthapavan, Fabrice Etilé, Adrian J. Cameron

**Affiliations:** 1 Deakin University, Geelong, Global Obesity Centre, Institute for Health Transformation, Locked Bag 20000, Victoria 3220 Geelong, Australia; 2 Biostatistics Unit, Deakin University, Locked Bag 20000, Geelong, Victoria 3220 Australia; 3 City of Greater Bendigo, PO Box 733, Bendigo, Victoria 3552 Australia; 4 Deakin Health Economics, Institute for Health Transformation, Faculty of Health Deakin University, Locked Bag 20000, Geelong, Victoria 3220 Australia; 5 National Institute for Health Innovation, University of Auckland, Private Bag 92019, Auckland, 1142 New Zealand; 6 Paris School of Economics and INRA, 48, Boulevard Jourdan, 75014 Paris, France

**Keywords:** Supermarket, Intervention, Shelf tag, Signage, Process evaluation, Perceptions, Consumer, Australia

## Abstract

**Background:**

Successful implementation and long-term maintenance of healthy supermarkets initiatives are crucial to achieving potential population health benefits. Understanding barriers and enablers of implementation of real-world trials will enhance wide-scale implementation. This process evaluation of a healthy supermarket intervention sought to describe (i) customer, retailer and stakeholder perspectives on the intervention; (ii) intervention implementation; and (iii) implementation barriers and enablers.

**Methods:**

*Eat Well @ IGA* was a 12-month randomised controlled trial conducted in 11 Independent Grocers of Australia (IGA) chain supermarkets in regional Victoria, Australia (5 intervention and 6 wait-listed control stores). Intervention components included trolley and basket signage, local area and in-store promotion, and shelf tags highlighting the healthiest packaged foods. A sequential mixed-methods process evaluation was undertaken. Customer exit surveys investigated demographics, and intervention recall and perceptions. Logistic mixed-models estimated associations between customer responses and demographics, with store as random effect. Supermarket staff surveys investigated staff demographics, interactions with customers, and intervention component feedback. Semi-structured stakeholder interviews with local government, retail and academic partners explored intervention perceptions, and factors which enabled or inhibited implementation, maintenance and scalability. Interviews were inductively coded to identify key themes.

**Results:**

Of 500 customers surveyed, 33%[95%CI:23,44] recalled the *Eat Well @ IGA* brand and 97%[95%CI:93,99] agreed that IGA should continue its efforts to encourage healthy eating. The 82 staff surveyed demonstrated very favourable intervention perceptions. Themes from 19 interviews included that business models favour sales of unhealthy foods, and that stakeholder collaboration was crucial to intervention design and implementation. Staff surveys and interviews highlighted the need to minimise staff time for project maintenance and to regularly refresh intervention materials to increase and maintain salience among customers.

**Conclusions:**

This process evaluation found that interventions to promote healthy diets in supermarkets can be perceived as beneficial by retailers, customers, and government partners provided that barriers including staff time and intervention salience are addressed. Collaborative partnerships in intervention design and implementation, including retailers, governments, and academics, show potential for encouraging long-term sustainability of interventions.

**Trial registration:**

ISRCTN, ISRCTN37395231 Registered 4 May 2017.

**Supplementary Information:**

The online version contains supplementary material available at 10.1186/s12966-021-01104-z.

## Background

Supermarkets are the leading source of food and beverage purchases globally [1] and are therefore a major influence on population diet and health [2]. The small number of in-store supermarket interventions designed to promote healthier diets (‘healthy supermarket initiatives’) conducted to date have demonstrated short-term potential to improve the healthiness of consumer purchases by changing aspects of the ‘4Ps’ of marketing: Product, Promotion, Place [3] and/or Price [4, 5] to favour healthier foods and beverages. Population health benefits require such changes to be maintained in the long-term. Understanding the barriers and enablers of successful implementation and maintenance can provide useful information for wide-scale implementation of healthy supermarket initiatives and therefore should be a research priority [6].

The sustainability of healthy supermarket interventions refers to both the maintenance of the intervention and capacity to sustain intervention effects into the future [7]. This may depend on a number of interrelated factors associated with the interventions themselves and the context within which they are implemented. Process evaluations of previous supermarket and grocery store interventions [8–11] have identified barriers to implementation and sustainability that include excessive retailer staff time and resource requirements [9], whereas enablers included perceived high customer satisfaction [8, 9] and improved retail brand image [9].

We are not aware of any mixed-methods process evaluations of supermarket initiatives that have considered the perspectives of customers, staff and other key stakeholders together. Critically, previous supermarket interventions and related process evaluations have generally been conducted 3 to 6 months into a project [8, 9, 11]. Process evaluations of supermarket interventions over the longer-term are essential to determine the potential for real-world sustainability and scalability, which relies on changes being maintained and adapted at scale over many months or years.

Here, we conduct an evaluation during and following a 12-month supermarket healthy eating intervention, ‘*Eat Well @ IGA*’ which aimed to improve the healthiness of customer purchases and increase sales of healthier packaged products and fresh produce. The process evaluation sought to investigate the experiences of customers, staff and stakeholders involved in *Eat Well @ IGA* development and implementation. The objectives were to (i) describe customer, retailer and stakeholder perspectives of the *Eat Well @ IGA* intervention; (ii) describe intervention implementation and any variation across intervention sites; and (iii) explore barriers and enablers of implementation.

## Methods

### Overview

**Fig. 1:**
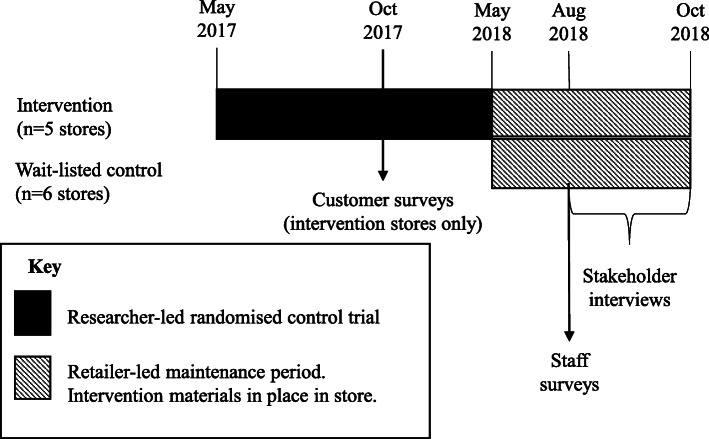
Timeline of *Eat Well @ IGA* randomised controlled trial and process evaluation

An overview of the development, timelines, and methods used in the *Eat Well @ IGA* project [12] has been previously published [13]. The timeline specific to the process evaluation is shown in Fig. 1.

Briefly, the *Eat Well @ IGA* project began in 2015 as a series of three short-term retailer-conceived healthy supermarket trials, implemented and evaluated in collaboration with local government and academic partners. The *Eat Well @ IGA* Randomised Controlled Trial (RCT) was a 12-month, multi-component intervention conducted between May 2017 and May 2018 in 11 Independent Grocers of Australia (IGA) supermarket stores in regional Victoria, Australia (5 intervention and 6 wait-listed control stores). Stores were recruited for RCT participation via the supermarket advertising collective of seven store owners (total 11 stores), which included the supermarkets that participated in the initial short-term trials. All stores agreed to participate in the intervention and evaluation.

The intervention included three components: (i) signage (trolley and basket signs, floor signs, aisle fins, healthy food posters, and shelf signs (“wobblers”)); (ii) local area and in-store promotion (flyers (mailers and in-store), social media, and launch event and related media); and (iii) shelf tags highlighting (healthiest) packaged foods store-wide (defined as all products with 4.5 or 5 stars according to the voluntary government-endorsed Australian and New Zealand front-of-pack Health Star Rating (HSR) system [14]). The process evaluation of the multi-component intervention is the focus of the current paper. A summary of the results of the *Eat Well @ IGA* RCT on supermarket sales has been published separately [13]. Briefly, preliminary results based on multi-level modelling show that the *Eat Well @ IGA* intervention was associated with slight significant increases in the sales of products with a Health Star Rating of 4.5 or higher, and in sales of fruits and vegetables, but had no significant effect on overall sales of healthy ‘core’ food according to the Australian Dietary Guidelines [15]. Retailers reported that there was no overall effect of the intervention on profit.

In addition, researchers collected intervention monitoring data to establish the fidelity of implementation (findings to be published in a separate paper alongside changes in supermarket sales). In summary, initial dose of intervention components varied across stores, mainly due to variation in store size. HSR shelf tags and trolley and basket signs were maintained at close to 100% fidelity throughout the intervention period. Display of posters, aisle fins and shelf wobblers initially increased as store staff gradually implemented all the signage, and then decreased from October 2017 to the end of the official intervention period in May 2018.

A sequential mixed-methods process evaluation was undertaken that included customer surveys (October 2017), staff surveys (August 2018) and qualitative stakeholder interviews (August to October 2018) (Fig. 1).

Customer surveys were conducted in the five intervention stores during the trial period, and staff surveys and stakeholder interviews were conducted in both the five intervention stores and the six wait-listed control stores after completion of the trial (May 2018) when both intervention and wait-listed control stores were self-maintaining the intervention. We used findings from customer surveys to develop methods for subsequent staff surveys, and then used customer and staff survey findings to develop interviews (explained in detail below).

Constructs for investigation and selection of measurement tools were informed by: established process evaluation frameworks including RE-AIM [16]; the Diffusion of Innovations theory [17] which describes how an idea or innovation is spread and adopted within a social system and characteristics of interventions likely to spread; Baranowski and Stables’ methodological guide [6] to assessment of key process evaluation constructs; as well as the intervention’s program logic model [13].

### Customer surveys

#### Overview

Customer exit surveys were conducted at all five intervention stores approximately 6 months into the intervention (October 2017) to collect self-reported data on (i) intervention reach including customer demographics; (ii) recall of the intervention; (iii) perceptions of overall intervention, specific intervention components, and potential alternate or additional interventions (measured on seven-point Likert scales; 1 = ‘strongly dislike’, 7 = ‘strongly like’); and (iv) perceptions of overall intervention and individual component effectiveness in promoting healthier choices. Survey questions were developed based on constructs relevant to the specific intervention components, informed by previous retail customer surveys [18], and adapted from questions successfully piloted in the three previous *Eat Well @ IGA* short-term trials. See Additional file 1 for full customer survey.

#### Sampling and recruitment

Exiting customers at the five stores who were aged 18 years or older and the main grocery buyer in their household were invited to self-complete the approximately 10-min, anonymous, paper-based survey. Sample size was decided based on pragmatic considerations and considering the primary purpose of the survey that was to provide feedback to the retailers on customer satisfaction and intervention component recall. A sample of 100 customers per intervention store was considered large enough to estimate the prevalence of binary outcomes with 95% confidence intervals which in the worst case (50% prevalence) would have a 20% total width.

#### Analysis

Customer perceptions of intervention components and suggestions for additional healthy food initiatives at the survey store were dichotomised for analysis due to extreme skewness of the distribution of the responses to many questions (1 to 4 = unfavourable or neutral impression/ unsupportive or neutral support; 5 to 7 = favourable impression/ supportive). Customer demographics were also dichotomised: age (18 to 54 years; 55 years and older), gender (male; female), area-based socioeconomic status according to postcode (Socio-Economic Indexes for Areas (SEIFA) Index of Relative Socio-Economic Disadvantage (IRSD)) [19] (lower half of SEIFA percentiles; upper half of SEIFA percentiles), educational attainment (less than university degree; university degree or higher), and frequency of customer visits (never, rarely or sometimes shop at survey store; always or usually shop at store). Heterogeneity of customer demographics across stores was assessed using the Chi-squared test. Overall proportion of customers in each demographic group (and 95% CI), and associations between customers’ perceptions and customer demographic variables were estimated using mixed-effects logistic models with the demographic variable as a fixed effect and store as random effect to account for the clustering induced by store. The intracluster correlation coefficient (ICC) is reported as a measure of variability of customer recall across stores.

Results at *p* < 0.05 were considered statistically significant. Stata version 15 was used for all analyses.

### Staff survey

#### Overview

Anonymous staff surveys were self-completed in August 2018, 3 months after the end of the RCT intervention period during the retailer-led maintenance period to (i) investigate staff involvement with and attitudes towards the intervention, and (ii) assess perceptions of customer response to the intervention. Surveys took approximately 10 min to complete and included closed-ended and free-text questions on personal demographics, interactions with customers, and attitudes towards the overall intervention and its specific components. Questions from the full questionnaire are mapped to these concepts and to Baranowski and Stable’s process evaluation framework [6] in Additional file 2.

#### Sampling and recruitment

All staff within all 11 stores (including the 5 intervention and 6 wait-listed control stores) were eligible to participate. After receiving consent from store managers, flyers were displayed in staff rooms and/or notice boards inviting staff to complete the paper-based survey.

#### Analysis

Descriptive statistics were used to summarise findings and inform development of stakeholder interview questions.

### Stakeholder interviews

#### Overview

The first author (MRB) conducted semi-structured interviews with stakeholders from intervention and selected wait-listed control stores at 15–17 months following the start of the RCT (August to October 2018, during the retailer-led maintenance period in intervention and wait-listed control stores). Stakeholders were defined as those involved in co-designing and managing the intervention, and those responsible for intervention implementation. Interviews aimed to explore (i) stakeholder perceptions of the *Eat Well @ IGA* intervention; (ii) factors which enabled or inhibited intervention implementation and may affect maintenance and scalability; and (iii) explanations for customer and staff survey findings.

#### Interview questions

The 30–60-min interviews focused on interview participant roles, intervention advantages, disadvantages, barriers, enablers and sustainability, stakeholder communication, and potential improvements for the *Eat Well @ IGA* intervention*.* Interview questions were developed by the research team to address key concepts of the Diffusion of Innovations theory [17] and RE-AIM framework [16]. Questions are mapped to these concepts in the full discussion guide (see Additional file 3). Questions were tailored to stakeholder roles.

#### Sampling and recruitment

Interviewees were purposively selected given their likely depth of knowledge regarding different components of the intervention, lines of communication and relationships between intervention partners (academic, retail, and government bodies) (see Additional file 4 for lines of communication) and selected to explore predicted causes of heterogeneity in implementation between stores (e.g., customer demographic composition). It was estimated that interviewing 15 to 20 key stakeholders would cover the breadth and depth of perceptions relating to the intervention. The interviewees comprised the local government officer responsible for research-retailer liaison, one supermarket group marketing manager, the main store group chief executive officer, all RCT intervention store managers (five), two store managers from highest and lowest SEIFA control stores, four grocery and fresh produce department managers from intervention stores with the lowest and highest SEIFA, three researchers (research lead; the data manager; and a research assistant), and two store owners.

#### Analysis

Interviews were audio recorded and transcribed verbatim. Interview transcripts were sent to participants for verification; participants did not express any concerns and no changes were made. Inductive coding and a block and segment analysis approach were used to identify key interview themes and sub-themes using NVivo qualitative data management software [20]. Coding was initially completed by an experienced qualitative researcher (MRB). A subset of 11 interviews was independently cross-coded by a second experienced qualitative researcher (CZ), and both coders discussed findings to decide on final themes.

## Results

### Customer surveys

Five hundred customers completed surveys at the five intervention stores (range: 97–104 per store) in October 2017 (Table 1). Respondents were more likely to be female (72%), 55 years or older (65%), have a higher area-based socioeconomic status (SEIFA) (61%), and speak English at home (100%) compared to the Victorian population. Sixty-four percent of respondents always or usually did their main planned (e.g. weekly/fortnightly) shop at the survey store. There was heterogeneity across stores by whether customers conducted their regular weekly shop always or usually at the survey supermarket, educational attainment, and area-based socioeconomic groupings (SEIFA), reflecting the location of participating stores (all *p* ≤ 0.01).

**Table 1:** Demographic characteristics of customer survey participants (*n* = 500)

Characteristic	Overall		Store 1 (***n*** = 104)	Store 2 (***n*** = 97)	Store 3 (***n*** = 101)	Store 4 (***n*** = 100)	Store 5 (***n*** = 98)	Heterogeneity across stores (***p***-value) ^**b**^	State of Victoria (%)
	n	Estimated percentage [95%CI] ^**a**^	Proportion (%)						
Female	355	72 [68,76]	67	75	70	75	74	0.650	51.9 ^c^
Aged 55 + years	322	65 [61, 70]	63	68	65	72	60	0.445	33.0 ^c^
High SEIFA ^d^	284	61 [16, 93]	7	92	93	10	89	< 0.001	~ 50
University degree or higher	165	34 [26,42]	24	32	34	27	52	< 0.001	24.3 ^c^
Regular weekly shop always or usually at this store	234	64 [56,72]	67	71	60	73	49	0.010	N/A
Shop regularly at competitor supermarket	463	93 [90, 95]	92	92	90	93	96	0.621	N/A

*n* = 500 customer surveys at the 5 intervention stores, range of 97 to 104 surveys per store. *N/A* data not available

^a^ Estimated using univariate logistic models with store as random effect to account for the clustering induced by store. ^b^ Chi-squared test of differences in proportions across stores; ^c^ Persons 15 years and older; ^d^ SEIFA, Index of Relative Socio-Economic Disadvantage- Socio-Economic Indexes for Areas [19]. ‘High’ SEIFA reflects top half of Victorian SEIFA groupings

Thirty-three percent [95%CI:23,44] of respondents recalled the *Eat Well @ IGA* project brand overall when prompted (see Table 2). Generally, half or fewer respondents recalled each intervention component. The highest recall was for the healthy food posters (49%[95%CI:37,61]), and the lowest for social media (12%[95%CI:7,18]). Some customers reported that Health Star Rating (HSR) shelf tags (49%[43,56]), posters (49%[95%CI:37,61]), shelf signs (29%[95%CI:30,43]), and trolley and/or basket signs (18%[95%CI:14,23]), influenced their food choices. Of those who said that trolley and/or basket signs had influenced their purchases, 69%[95%CI:49,84] reported that signs increased purchases of foods which corresponded to the core food groups from The Australian Dietary Guidelines (grains; vegetables and legumes/ beans; fruit; lean meats and alternatives; and dairy and alternatives) [15]. ICC estimates for intervention component recall ranged from 0.009 (letterbox flyers) to 0.113 (staff t-shirts), suggesting variation across stores for some components (Additional file 5, Table S1).

**Table 2:** Customer recall of *Eat Well @ IGA* project components (*n* = 500), October 2017

Project component recall (estimated percentage [95%CI])	Overall ^a^ (n = 500)	Customer demographic subgroups ^b^									
		Age		Gender		SEIFA		Educational attainment		Store shopping frequency	
		18-54y (***n*** = 172)	55y and older (***n*** = 324)	Male (***n*** = 137)	Female (***n*** = 357)	Low (***n*** = 213)	High (***n*** = 285)	High school or lower (***n*** = 327)	University or higher (***n*** = 165)	Regular (***n*** = 136)	Infrequent (***n*** = 235)
*Eat Well @ IGA* overall	33 [23, 44]	37 [26, 49]	31 [22, 41]	**25 [16,37]**	**36 [26,48]**	35 [24,48]	31 [21,43]	32 [22,42]	37 [26,49]	35 [24,49]	25 [15,39]
Trolley and/or basket signs	44 [33,56]	50 [37,63]	42 [31,54]	41 [28,55]	46 [35,58]	38 [24,54]	49 [34,64]	42 [32,54]	48 [36,61]	**49 [34,64]**	**36 [23,53]**
HSR shelf tag	48 [40,57]	**56 [45,66]**	**45 [36,54]**	43 [32,54]	50 [41,59]	44 [32,57]	51 [40,62]	47 [39,56]	50 [40,61]	52 [40,63]	43 [31,56]
Posters	49 [37,61]	**64 [51,76]**	**42 [30,55]**	**41 [28,56]**	**52 [39,65]**	50 [35,65]	52 [35,62]	47 [35,59]	55 [41,68]	56 [43,69]	45 [32,60]
Shelf signs	37 [30,43]	**45 [37,54]**	**33 [27,40]**	30 [22,39]	40 [33,47]	35 [27,45]	37 [30,45]	36 [29,43]	39 [31,48]	41 [32,50]	30 [21,40]
Letterbox flyers	14 [10,17]	13 [9,20]	13 [10,18]	12 [8,20]	14 [10,19]	12 [7,20]	14 [10,21]	14 [10,18]	13 [8,19]	**16 [9,26]**	**6 [2,13]**
Staff t-shirts	24 [15,37]	26 [15,41]	24 [14,37]	23 [13,38]	25 [15,38]	23 [13,39]	25 [14,39]	25 [15,39]	23 [13,37]	28 [18,40]	25 [15,38]
Social media	12 [7,18]	13 [7,21]	11 [7,18]	10 [6,19]	13 [8,19]	14 [9,24]	10 [6,16]	12 [8,19]	10 [6,18]	13 [9,18]	12 [8,19]

All questions scored as Yes/No recall of components. Bolding indicates significant difference (*p* < 0.05) between subgroups. *HSR* Health Star Rating; *IGA* Independent Grocers of Australia; *SEIFA* Index of Relative Socio-Economic Disadvantage- Socio-Economic Indexes for Areas. ^a^ Estimated using logistic mixed-effects models with store as random effect to account for the clustering induced by store. ^b^ Estimated using logistic mixed-effects logistic models with the demographic variable as a fixed effect and store as random effect.

Customer perceptions were generally skewed towards more favourable ratings, with all intervention components receiving median ratings of 5 or 6 out of 7 (Additional file 5, Table S2).

Respondents overwhelmingly agreed that IGA should continue its efforts to encourage healthy eating (97%[95%CI:93,99]) (Table S3). Three percent [95%CI:1,6] of respondents reported they were new customers to the store because of the intervention and 7%[95%CI:5,9] reported they had increased their shopping frequency at the store as a direct result of the intervention (only one customer reported they were shopping less frequently).

#### Customer subgroup analyses results

Customer subgroup analyses are reported in Table 2 and Additional file 5 (Tables S2 and S3). Respondents 55 years and older were less likely to recall Health Star Rating (HSR) shelf tags (*p* = 0.03), posters (*p* < 0.01), and shelf signs (*p* < 0.01). Compared to infrequent shoppers, regular shoppers were up to twice as likely to recall the project overall (*p* = 0.05), trolley and basket signs (*p* = 0.03), shelf signs (*p* = 0.05) and letter box flyers (*p* < 0.01). There was no difference in project or component recall between gender, educational attainment or Socio-economic Index for Areas (SEIFA) levels (all *p* > 0.05).

Respondents 55 years and older generally rated their perceptions of study components more highly than younger respondents (18 to 54 years) (Table S2). Female respondents gave higher ratings to the intervention overall as well as most components than male respondents, as did shoppers with higher compared to lower socioeconomic position (all *p* < 0.05) (Table S2). Regular survey store shoppers rated all components more highly than infrequent shoppers (all *p* ≤ 0.03), except floor signs (*p* = 0.09) and social media (Table S2). There was no difference in perceptions of project components by educational status. All subgroups had a mean of at least 94% support for IGA continuing its efforts to encourage healthy eating (Table S3). All subgroups had a mean of at least 75% support for new interventions of healthier products at the end of aisles and availability of healthy recipes in-store (Table S3).

### Staff surveys

A total of 82 staff from 11 stores completed surveys in August to September 2018 (see Additional file 6, Table S4 for respondent demographics). No differences were found in staff characteristics between intervention and wait-listed control store surveys, hence we present overall results. The most frequent respondent role was department manager (29%). Eighty percent of staff had been employed in their particular store for 2 years or more, meaning that they had been employed prior to the implementation of *Eat Well @ IGA*. Most respondents (84%) reported that they had not been directly involved with installation or maintenance of *Eat Well @ IGA*.

Staff had very favourable perceptions of *Eat Well @ IGA* overall (median 6 [5, 7] on 7-point Likert scale) and specific intervention components (see Additional file 6, Table S5). The most frequently cited components that “worked well” were trolley/ basket signs (27% respondents), followed by HSR shelf tags (11%) and floor sign (11%), which staff reported to be the most noticeable by customers (Additional file 6, Table S6). The most frequently cited components that “did not work well” were the HSR shelf tags (17% respondents), followed by shelf signs (10%). The tags were noted to fall off easily. The shelf signs were noted to both fall off easily and get in the way of other products and signage (Additional file 6, Table S7). Most staff (78%) had rarely or never received feedback from customers relating to *Eat Well @ IGA* or its components (Additional file 6, Table S8). The majority of feedback received, however, was very or slightly positive (e.g. “sending a positive message to customers”), or otherwise neutral (e.g. “[customer surprised] at the low star rating of products”). Only one respondent reported receiving “slightly negative” customer feedback, and none reported “very negative” feedback.

The majority of staff (93%) thought *Eat Well @ IGA* would be maintained at their store in the future. Reasons for thinking the intervention would not be maintained varied but comments included “It’s not maintained now either”, and that profit would be the main driver of supermarket strategies (e.g. “[price] specials sell first”). Respondents were asked to rank seven additional healthy eating strategies from least to most effective in terms of whether they would help customers make healthy choices (Additional file 6, Table S9). The highest median ranked option was “Price discounts on healthy foods and drinks” (2 [1, 3] out of 7). The lowest ranked was “One checkout that doesn’t display unhealthy food” (7 [6, 7]).

### Stakeholder interviews

**Fig. 2:**
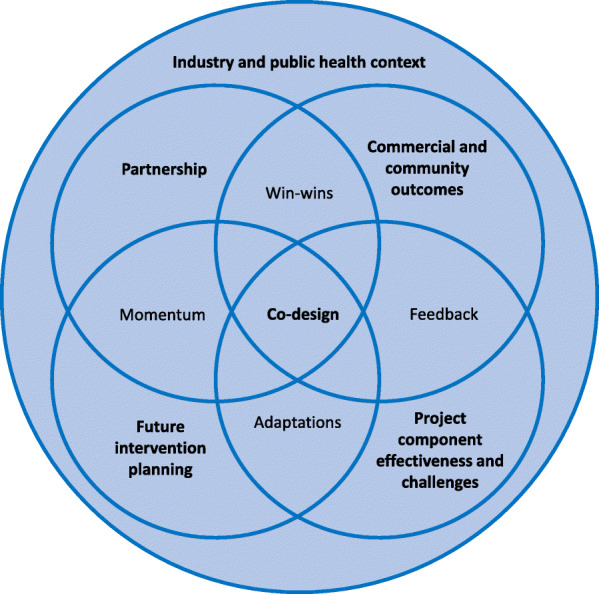
Interrelationships between stakeholder interview themes on factors that were perceived to enable or inhibit the implementation, maintenance and scalability of Eat Well @ IGA, August to October 2018 (*n* = 19 interviews). Bolded text indicates main interview themes and unbolded text indicates interrelationships between themes

Nineteen stakeholders participated in interviews between August and October 2018 (95% response rate). Seventy-nine percent of interviewees were male, and all had been in their current role for at least 6 months. Below, key themes are discussed (theme and sub-theme names in *italics*). The themes encompass stakeholder perceptions of the intervention including factors which enabled or inhibited intervention implementation and may affect maintenance and scalability, as well as the perceived intervention outcomes. The interrelationship between these themes are shown in Fig. 2, with stakeholder collaboration and co-participation in planning, implementation and evaluation of the intervention (the “Co-design” approach) central to the project. A full table of themes and sub-themes is found in Additional file 7.

#### Theme 1: Consider the influence of industry and public health context on intervention

Interviewees described multiple contextual factors that influenced the implementation of *Eat Well @ IGA *– namely, the broader supermarket industry context (defined as traditional business operations focused around sales and profitability) and the public health context (defined as the current health issues experienced by the target communities). Competing ideas and priorities were often alluded to as potential barriers and enablers to intervention implementation, within each of these sub-themes.

From an industry perspective, standard *Supermarket operations* were generally seen as a hindrance to implementing and sustaining healthy changes as business models tend to favour promoting sales of unhealthy foods and beverages because of perceived consumer demand and supplier contract agreements (e.g. *Status quo is unhealthy; Power of conglomerates*). However, broader *Industry change* was occurring in response to *Customer trend*s towards healthy eating, particularly by health-conscious consumers and those with children; this increased the retailers’ interest in implementing *Eat Well @ IGA*.

“I think the whole market's starting to change… people gravitating towards all those healthier type products. So I think within a couple of years, the demand for those products – well, it's already there now, but it's going to be even stronger.” (Store Manager).

The public health benefit of promoting healthy eating through *Eat Well @ IGA* was expressed by some store owners who viewed the intervention as *Responding to community health needs* based on their community context of poor health outcomes or deprivation:

“…our demographics around here, it's probably - if you just make people make one [healthier] choice differently, it's a winner that way.” (Store Manager).

The researchers and local government research officer viewed the project as having a broader contribution to *Public health advocacy* beyond the immediate community, through the potential scale-up of *Eat Well @ IGA* through the IGA network and diffusion to other supermarket chains.

#### Theme 2: Develop project partnership and align stakeholder objectives

The *Partnership*, referring to the people and interpersonal relationships between academic, IGA, local government, and (to a lesser extent) external funder stakeholders, underpinned the conceptualisation, planning and implementation of the intervention. *Partnership* was more explicitly valued by those involved in setting up the intervention: the research lead and the local government research officer placed high emphasis on *Stakeholder relationships* being pivotal to the implementation and maintenance of *Eat Well @ IGA*, based on high levels of trust and *Collaboration* over several years. These stakeholders also placed greater emphasis on the *Credibility* of the project that was gained through local government and university participation, and government funding; and the necessity of *Expertise* in areas of nutrition and research (researchers), and supermarket operations (IGA executives). By contrast, store managers and department managers focused their discussions on the practicalities of day-to-day implementation of the intervention, although many acknowledged the practical and strategic value of the partnership. The *Partnership* was perceived by interviewees to be strengthened by *Aligning stakeholder objectives –* specifically relating to promoting healthy eating and business sales.

“One, the whole objective of the campaign was to try and influence or encourage people to eat healthier but also it was a win/win in the means of if we’re selling more of those 4.5, 5 star [Health Star Rating] type products within our stores there’s the potential of … higher profit…” (IGA Executive).

#### Theme 3: Co-design and collaborate for intervention design and progression

Stakeholders were universal in their praise for the value of the process through which stakeholders in the *Partnership* (described above) collaborated and co-participated to design and progress the intervention, a process which we term “*Co-design*”. For example, stakeholders described the productive process of working together in *Planning* and *Selecting intervention components:*

“So we sort of all sat down and discussed a range of options for what we could try in those stores and together we decided on the Health Star Rating shelf tags, the trolley signs and…the floor signs.” (Local Government Research Officer).

Timely *Feedback* was considered important to the supermarket executives to enable decision-making to proceed and to determine how the project should move forward, but this was limited by contrastingly long research timeframes in applying for *Funding*, and data analysis and publication.

#### Theme 4: identify intervention component characteristics, effectiveness and challenges

Stakeholders expressed mixed views when asked about the effectiveness, challenges and proposed modifications of specific project components. Store managers who believed that the intervention would be more effective appeared to be more engaged in intervention maintenance and scale-up. Comments on specific components were similar to staff survey feedback (see above). Participants most commonly proposed *Modifications* to overcome issues with *Maintenance* of *HSR shelf tags.* For example, in one store:

“they'd [the supermarket staff] basically made their own tape or data strip tape to actually sit inside [the shelf strips], … a general *Eat Well @ IGA* one.” (Researcher).

Other general concerns about intervention components included that the *Salience of point-of-sale material decreases over time* (in the context of ubiquitous marketing causing *Visual noise in the supermarket)*:

*“*we sometimes use the term ‘air pollution’, because sometimes it's just too busy and people don’t stop and read. But the baskets and the trollies; it's basically right in the customer’s face.” (Store Manager).

Several supermarket stakeholders recommended regularly refreshing the marketing material to maintain intervention effectiveness.

#### Theme 5: Consider the commercial and community outcomes

The main intervention outcomes raised by participants broadly fell into (i) commercial or profit-related outcomes, and (ii) community outcomes related to customer feedback and purchasing effects. *Profit impact* was generally predicted to be either neutral (given that supermarkets mostly *Profit from unhealthy foods*) or to slightly increase (as *Profit margins are higher on healthy products* including fresh produce).

“we've got to put a chocolate [on display at the checkout] because we've got to survive, we've got to make money to keep our staff employed and pay our bills.” (IGA Executive).

*Return on investment* was a central consideration of executives and store owners for determining commercial viability of the intervention:

“the question for us as a business is what's it going to cost and then what's the return on it?” (IGA Executive).

Rather than direct profit returns, most returns were discussed in light of *Competitive advantage* by improving *Corporate image* and creating a point-of-difference among competitor supermarkets. The majority of investment/cost discussions related to staff *Time as a resource* to maintain the intervention, particularly the HSR shelf tags. Most store and department managers estimated 1–4 staff hours would be required to maintain the tags per month, although the perceived value of this time varied. A smaller sub-theme was *Customer feedback*. Customers were generally noted to have had a low awareness of the intervention and this therefore did not appear to affect intervention outcomes.

#### Theme 6: Plan and adapt for the future using evidence and support

When asked about the future sustainability and scalability of *Eat Well @ IGA,* interviewees outlined the need for evidence, funding, practical support and improvements in some components*.* Stakeholders noted that *Sustainability* of the intervention in current stores and *Scale-up* to other IGAs or supermarket chains would need to be underpinned by *Evidence* of effectiveness (thought to be *Uncertain* prior to rigorous quantitative evaluation) and *Ongoing modification* to refine intervention components. The vast majority of intervention installation and maintenance was done or paid for by the researchers. Some supermarket and non-supermarket interviewees expressed concern that removal of this assistance would make intervention *Sustainability* less likely. *Scale-up* was almost universally seen as desirable, but limited by some concerns that without researcher or other central oversight it would not be enforced or implemented ‘correctly’ to improve the healthiness of customer choices, rather than as a marketing tool to create a perception of being a healthier place to shop compared to competitors.

“I think if the results come back are good and everything’s positive I think the potential’s huge more so from a macro or IGA brand.” (IGA Executive)

## Discussion

This process evaluation of the complex supermarket intervention *Eat Well @ IGA* demonstrated that it had high support from customers, staff and broader stakeholders. Key learnings included barriers around staff time required for intervention maintenance and perceptions of reduced salience of the intervention over time among customers due to the ubiquitous marketing noise in the modern retail environment. The collaborative partnership and co-participation between stakeholders – what we have termed “co-design” – was a key enabler of planning, implementation and future directions of the project.

Findings from this study align with previous evaluations of supermarket and grocery store interventions [11], including the identification of staff time as a key barrier to sustainability [9, 21]. Our stakeholder interviews have extended these previous studies by revealing that these concerns persisted at 12 months. Key concerns related to the opportunity cost of staff time (i.e. staff time spent on *Eat Well @ IGA* instead of other projects) and the expected return on investment of the time and effort involved in intervention implementation, rather than the financial cost or number of staff hours required per se, which was estimated around 1–4 h per month.

Our use of the Diffusion of Innovations theory [17] and Baranowski and Stables’ process evaluation guide [6] in survey and interview question design helped to provide insights into whether or not *Eat Well @ IGA* embodied the usual characteristics of scalable innovations. For example, interviewees expressed conflicting views on the Diffusion of Innovation [17] characteristic of “compatibility” of the intervention with status quo supermarket operations. On the one hand, most stakeholders acknowledged that status quo operations tend to favour unhealthy foods and beverages. On the other hand, some stakeholders including retailers expressed hope that it might address perceived increasing consumer demand for healthier supermarket offerings. Senior IGA stakeholders discussed advantages (e.g. potential profit increases) and disadvantages (e.g. increased staff time) of the intervention in relation to current practice (described by the Diffusion of Innovations theory as “Relative advantage” [17]). Future monitoring of IGA networks will be required to determine whether *Eat Well @ IGA* does in fact diffuse to other IGA stores.

Low customer recall of the *Eat Well @ IGA* brand (33% [95%CI:23, 44]) and project components was indicated by both customer surveys and noted in some stakeholder interviews. Staff survey results suggested that salience of the intervention decreased over time. As such, promotional material may need to be periodically refreshed to remain effective and to cut-through the visual noise in the supermarket [22]. The issue of salience decreasing over time has not been a key finding of other relevant process evaluations, perhaps due to the shorter timeframe of previous evaluations, and the modest size of the current merchandising-based intervention. Alternatively, the effectiveness of the intervention may not just depend on recall, but rather the intervention may work by a variety of psychological mechanisms. For example, many of the trolley and shelf signs targeted social norms around healthy eating. Future healthy food retail interventions that utilise marketing messages should plan for regularly refreshing campaign materials, while considering intervention cost and sustainability, and targeting different psychological pathways of effect.

Another novel component of our evaluation was examination of variation in customer recall and perceptions of components across stores. We found moderate variation in recall across stores, which may reflect extent of implementation. Higher recall of HSR shelf-tags, posters and shelf-signs was found among younger compared to older respondents in the customer survey. This may be related to age-related cognitive declines in attention, and aligns with stakeholder interviews which suggested that younger, health-conscious consumers (especially those with young families) engaged more with the project.

We found customers demonstrated extremely high self-reported support for *Eat Well @ IGA* and further changes to improve the healthiness of the in-store supermarket environment; however, staff and stakeholders reported limited customer feedback (positive or negative). A recent review of business outcomes in healthy food retail initiatives found that 65% of measured customer perception outcomes were favourable [18]. While customer perceptions were not a focus of stakeholder interviews, positive impacts on brand image [9] were central to supermarket stakeholder’s assessment of commercial viability. This was aligned with the IGA chain brand slogan “how the locals like it” and supermarket stakeholders’ references to industry growth in healthy food sales in Australia, which has also been recently identified in interviews with US supermarket retailers [21].

Customers expressed support for other potential changes to encourage healthier purchases, including healthier end of aisle displays and the availability of healthy recipes. This is consistent with other evaluations of similar interventions [10, 11]. Accordingly, these intervention components should be considered as part of future initiatives. Nonetheless, there are likely to be important barriers to implementing these suggestions as end of aisle displays are frequently dictated by contractual agreements with manufacturers, as highlighted by several interviewees.

The most important intervention outcomes specified by interviewees varied according to retailer role, with store staff focusing on staff resourcing needed for day-to-day intervention maintenance, and store managers and executives focused on community health outcomes and brand image. The strongest support for *Eat Well @ IGA* was expressed by stakeholders who prioritised the value of the intervention on community health outcomes. Whether the staff from smaller, community-based supermarkets such as IGA rate community health and goodwill more highly than staff from larger chain supermarkets (who often have less direct relationships with their local community) could not be determined in the current study. Moreover, suppliers were not identified by interviewees as a key barrier to implementation, despite being raised in previous interviews with supermarket [21] and small grocery store managers [23]. This was likely because the current intervention did not involve changes in product placement or availability.

Considerable emphasis was placed on the value of the co-design approach between retailers, government and academic partners by those directly involved in the project development. Perceived advantages included the diverse attributes that different partners brought to the project including the credibility provided by local government and university participation, government funding, nutrition and research skills, and supermarket operations knowledge. Consistent with other community health promotion partnerships, our stakeholders identified challenges of academic and funding timelines [24], but also the dividends including sustainability brought by time investment in partnerships. Our findings were also in line with other supermarket-academic partnerships which have noted that co-design elements in planning, implementation and evaluation have been essential to programme sustainability [25].

There was strong support among the three main stakeholder groups (local government, supermarket, academic partners) for the partnership and *Eat Well @ IGA* to continue, provided modifications were made regarding HSR tags and refreshing of the materials, which could otherwise hinder intervention uptake. Some supermarket senior stakeholders required a profit increase directly attributable to the intervention to continue to support the project, while for most a neutral sales effect was sufficient given the perceived benefits to brand image and community wellbeing. There was uncertainty among interviewees about what the next steps should be, noting organisational and resourcing difficulties with scale-up to other IGAs. A 2016 systematic review found no evidence examining the scale-up of a supermarket intervention [3]; addressing this gap will be critical to increasing the population impact of healthy food retail initiatives, which must be maintained long-term across many settings to drive meaningful population health changes.

### Strengths and limitations

This research used a theory-driven [6, 16, 17] sequential mixed-methods design to examine a complex supermarket intervention from the complementary perspectives of different stakeholders. Demonstrating the value of the mixed-methods approach, qualitative and quantitative data sources were sometimes concordant (e.g. customer intervention recall), and at other times discordant (e.g. customer intervention support in customer surveys was not reflected in staff surveys). The 12-month time frame of the intervention provided a longer-term perspective on maintenance and sustainability of supermarket initiatives. Views of customers and staff surveyed may not be representative of the target population groups, as it is possible those who had more extreme views (positive or negative) were more likely to respond to surveys. Although we did not have data on all customer demographics, we found that surveyed customers tended to be older and to have a higher socioeconomic position than the general population (and therefore likely customers overall). The views of stakeholders may also change over time, noting that some stakeholders (retail and other) had been involved in healthy eating initiatives for up to 4 years prior to this evaluation. Repeated longitudinal assessment of the opinions of retail stakeholders throughout all phases of the intervention process would be of interest in future initiatives.

## Conclusions

This process evaluation of the 12-month *Eat Well @ IGA* supermarket RCT found that the retailer-academic-government partnership and co-design process was critical for the development and maintenance of this intervention. These findings are an important accompaniment to the outcome data on the effectiveness of the intervention, and will aid in interpretation of those findings. Our learnings emphasised the importance of limiting retailer time and costs and the need for periodic refreshment of point-of-sale marketing materials to maintain customer salience. Identifying and implementing factors that lead to long-term sustainable and widespread healthy supermarket initiatives is critical to realising the potential population health benefits of healthy supermarket initiatives.

## Supplementary Information

**Additional file 1.** Customer survey questions. Full list of customer survey questions.

**Additional file 2.** Supermarket staff survey questions. Full list of staff survey questions and target construct(s) per question.

**Additional file 3.** Stakeholder interview discussion guide. Stakeholder interview discussion guide and target construct(s) per question.

**Additional file 4.** Stakeholder roles and lines of communication. Figure showing lines of communications between different stakeholders, their places within organisations and main organisational roles.

**Additional file 5.** Customer survey detailed results. Detailed customer survey results including tabulated overall and subgroup analyses and text descriptions.

**Additional file 6.** Staff survey detailed results. Detailed staff survey results including tabulated overall and subgroup analyses.

**Additional file 7.** Stakeholder interview themes, sub-themes and illustrative quotes. Stakeholder interview themes, sub-themes and illustrative quotes.

## Data Availability

The data from the anonymous staff and customer surveys are available from the authors on reasonable request. The interview data are not publicly available due to containing sensitive personal information.

## Abbreviations

HSR: Health Star Rating
ICC: Intra-cluster correlation coefficient
IGA: Independent Grocers of Australia
IRSD: Index of Relative Socio-Economic Disadvantage
RCT: Randomised Controlled Trial
SEIFA: Socio-Economic Indexes for Areas
