# Supplementary material for: The ‘Eat Well @ IGA’ healthy supermarket randomised controlled trial: process evaluation
Source: Int J Behav Nutr Phys Act. 2021 Mar 12;18:36. doi: 10.1186/s12966-021-01104-z (PMC7953771; doi:10.1186/s12966-021-01104-z)
Supplement: Supplementary file 1 — Additional file 1. Customer survey questions. Full list of customer survey questions. [file 12966_2021_1104_MOESM1_ESM.docx]

**Additional file 1: Customer survey questions**


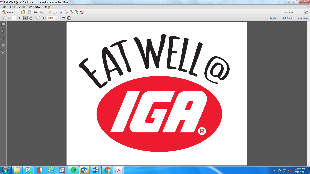

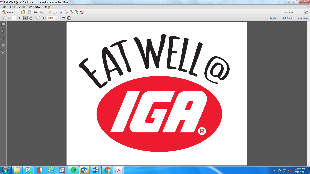
***Eat Well @ IGA* – Helping Create Healthier Communities**

**Tell us what you think!**

IGA are working with the **City of Greater Bendigo**, **VicHealth** and **Deakin University** to investigate ways to encourage healthy eating. As part of the ‘*Eat Well @ IGA*’ project, we are interested in finding out if you have noticed the changes we have made to this IGA store, what you think of the ‘*Eat Well @ IGA*’ project, and whether this has changed what you buy.

For further information about this study, please contact Dr Adrian Cameron ([adrian.cameron@deakin.edu.au](mailto:adrian.cameron@deakin.edu.au)).

Please read the Plain Language Statement and Consent document that contains more information about this project prior to participating.

To familiarise yourself with the ‘*Eat Well @ IGA*’ materials mentioned in this survey (shelf tags and signs, trolley signs, floor signs, posters) please look at the examples provided before completing this survey.

1. **Eligibility question: Are you over 18 and the main grocery buyer in your household?** *(Please circle)*
2. Yes - I do all or most of the shopping
3. Yes - I share the shopping
4. No - someone else does all or most of the shopping **(sorry, but we are only collecting information from main shoppers – you are not eligible to continue with this survey)**
5. How often do you do the following types of shopping at this IGA store? *(Please tick)*

|  | Always | Usually | Sometimes | Rarely or never |
| --- | --- | --- | --- | --- |
| My main planned (e.g weekly/fortnightly) shop. |  |  |  |  |
| To buy food for 1 or 2 days only |  |  |  |  |
| Unexpected/emergency shop |  |  |  |  |
| To only (or mainly) buy items on special in the weekly catalogue |  |  |  |  |

1. Thinking about the various supermarket chains, for each chain please indicate how often you shop there. *(Please tick)*

|  | Regularly  shop there | Occasionally  shop there | Rarely or never shop there |
| --- | --- | --- | --- |
| IGA |  |  |  |
| Coles |  |  |  |
| Aldi |  |  |  |
| Woolworths |  |  |  |

*Please circle Yes or No for the following questions*

1. Before this questionnaire, were you aware of the ‘*Eat Well @ IGA*' project? Yes / No
2. Do you recall receiving a flyer promoting ‘*Eat Well @ IGA*’ in your letterbox? Yes / No
3. Have you noticed staff in this store wearing T-shirts promoting ‘*Eat Well @ IGA*’? Yes / No
4. Have you noticed promotion through social media for ‘*Eat Well @ IGA*’? Yes / No
   1. If yes, which of the following have you noticed *(tick all that apply):*

❑ Facebook

❑ Twitter

❑ Instagram

1.
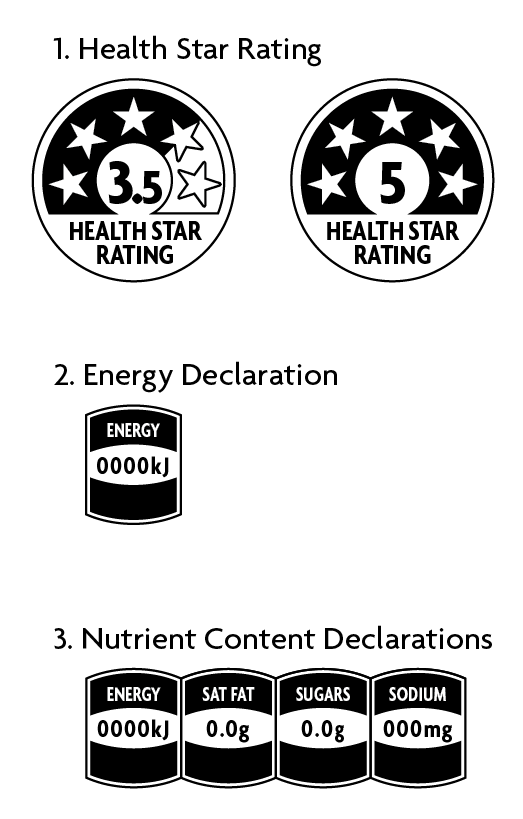
Are you familiar with the new Health Star Rating System being introduced on the front of pack of many products? Yes / No
2. Have you noticed shelf labels for all products with a 4.5 or 5 Health Star Rating in this store?

Yes / No **(go to Q11)**

1. If yes, do you believe these shelf labels influence what you purchase from this store? Yes / No / Unsure
2. Have you noticed other ‘*Eat Well @ IGA*’ signs on the shelves with healthy messages about specific products?

Yes / No **(go to Q12)**

1. If yes, do you think the shelf signs have influenced what you purchased from this store? Yes / No / Unsure
2. Have you noticed posters throughout the store promoting healthy choices and fresh fruit and vegetables?

Yes / No **(go to Q15)**

1. If yes, do you think these posters have influenced what you purchased from this store? Yes / No / Unsure
2. Have you noticed ‘*Eat Well @ IGA*’ signs in trolleys and baskets in this store?

Yes / No **(If no, skip to question 17)**

1. Do you believe that the trolley and/or basket signs influenced what you purchased from this store in the last month?

Yes / No / Unsure

1. If you answered yes, what types of products were you more or less likely to purchase?

______________________________________________________________________________________________________________________________________________________________________________________________

1. What was your overall impression of these ‘*Eat Well @ IGA*’ components?: *(please circle one number per question)*
2. Trolley and basket signs

Strongly dislike 1------2------3------4------5------6------7 Strongly like N/A

1. Floor signs

Strongly dislike 1------2------3------4------5------6------7 Strongly like N/A

1. Health Star Rating shelf tags

Strongly dislike 1------2------3------4------5------6------7 Strongly like N/A

1. Posters

Strongly dislike 1------2------3------4------5------6------7 Strongly like N/A

1. Shelf signs

Strongly dislike 1------2------3------4------5------6------7 Strongly like N/A

1. Letter box flyers

Strongly dislike 1------2------3------4------5------6------7 Strongly like N/A

1. Social media (Facebook, Instagram etc.)

Strongly dislike 1------2------3------4------5------6------7 Strongly like N/A

1. The ‘*Eat Well @ IGA*’ project as a whole

Strongly dislike 1------2------3------4------5------6------7 Strongly like N/A

1. Please let us know if you think that this store should include the following: *(please circle one number per question)*
2. One checkout that doesn’t display unhealthy food

Strongly disagree 1----2----3----4----5----6----7 Strongly agree

1. All checkouts don’t display unhealthy food

Strongly disagree 1----2----3----4----5----6----7 Strongly agree

1. Healthier products on display at the ends of aisles

Strongly disagree 1----2----3----4----5----6----7 Strongly agree

1. Healthy recipes

Strongly disagree 1----2----3----4----5----6----7 Strongly agree

1. Do you agree that IGA should continue its efforts to encourage healthy eating?

Strongly disagree 1------2------3------4------5------6------7 Strongly agree

1. Does the ‘*Eat Well @ IGA*’ project make you more likely to shop at IGA (rather than other supermarket chains)?

Strongly disagree 1------2------3------4------5------6------7 Strongly agree

1. Do you have any other feedback regarding ‘*Eat Well @ IGA*’ (positive or negative, how we could improve etc.)?

________________________________________________________________________________________________________________________________________________________________________________________________________________________________________________________________________________________________

1. Are you: *(Circle)* Male / Female / Other
2. How old are you: *(Circle)* 18-24 years 35-44 years 55-64 years

25-34 years 45-54 years 65+ years

1. Do you speak English at home? *(Circle)* Yes / No
2. What is your home postcode? ­­­­­­­­_________­
3. What is the highest level of education you have completed?

|  | Did not complete high school |
| --- | --- |
|  | Year 12 or trade certificate or diploma |
|  | University degree or higher |

1. Today’s date: _________________________
2. Store: *(tick a box)*

[list of surveyed stores]

1. Since the ‘*Eat Well @ IGA*’ project started in May 2017, do you shop at this IGA store more or less because of the ‘*Eat Well @ IGA*’ project? *(Circle)*

Less often / No change / More often

1. Are you a new customer to this IGA store since May 2017 because of the *Eat Well @ IGA* project? i.e. you decided to shop at this store because of their commitment to healthy eating *(Circle)*

Yes / No / Unsure
