# Supplementary material for: The ‘Eat Well @ IGA’ healthy supermarket randomised controlled trial: process evaluation
Source: Int J Behav Nutr Phys Act. 2021 Mar 12;18:36. doi: 10.1186/s12966-021-01104-z (PMC7953771; doi:10.1186/s12966-021-01104-z)
Supplement: Supplementary file 2 — Additional file 2. Supermarket staff survey questions. Full list of staff survey questions and target construct(s) per question. [file 12966_2021_1104_MOESM2_ESM.docx]

**Additional file 2: Supermarket staff survey questions**

| **No.** | **Question** | **Construct/ variable (not displayed in survey)** |
| --- | --- | --- |
|  | Which of these best describes your main job at IGA?   1. Store manager 2. Department manager 3. Work on the floor (restocking shelves etc. in Longlife/ Perishables/ Produce/ Liquor) 4. Work in deli/meat 5. Work at checkout 6. Work in administration 7. Other (please describe)______________ | Demographics |
|  | Is your employment at IGA:   1. Full-time 2. Part-time 3. Casual | Demographics |
|  | How long have you worked for IGA?   1. Less than 6 months 2. 6 months to 1 year 3. More than 1 year and less than 2 years 4. 2 years or more | Demographics- have they been around since before ‘*Eat Well @ IGA*’? |
|  | How often do your main planned (e.g. weekly/fortnightly) shop at IGA?   1. Always 2. Usually 3. Sometimes 4. Rarely or never 5. Not applicable- never the main shopper for the household | Staff as customers |
|  | What was your overall impression of these ‘*Eat Well @ IGA*’ components?: (please circle one number per question)  Strongly dislike 1---2---3---4---5---6---7 Strongly like Did not notice   1. Trolley and basket signs 2. Floor signs 3. Health Star Rating shelf tags 4. Posters 5. Shelf signs 6. Flyers 7. Social media (Facebook, Instagram etc.) 8. The ‘*Eat Well @ IGA*’ project as a whole | From customer survey  Staff attitudes  *Images of each component shown to participants* |
|  | Have you been involved with ‘*Eat Well @ IGA*’ with installation or maintenance of any of the following components? (Floor signs, Health Star Rating shelf tags, Posters, Shelf signs)   1. No 2. Yes- please describe_______________ | Intervention components (staff involvement/ exposure) ^a^ |
| **6b** | Please describe any of these components you thought worked particularly well and why.  (free text) | Enablers ^a^ |
| **6c** | Please describe any of these components you thought did not work and why.  (free text) | Barriers ^a^ |
| **6d** | Do you have any suggestions for how ‘*Eat Well @ IGA*’ or its components could be improved? | Adaptations to initial plans |
|  | How frequently have you had feedback from or conversations with customers about ‘*Eat Well @ IGA*’ or its components since ‘*Eat Well @ IGA*’ was introduced in your store?   1. Always 2. Usually 3. Sometimes 4. Rarely or never | Interactions with customers |
|  | Overall would you say the feedback from customers has been:  Very positive  Slightly positive  Neutral  Slightly negative  Very negative  Not applicable- no feedback received from customers | Interactions with customers |
| **7b** | If yes, can you describe any specific positive or negative customer feedback?  *(Free text entry)* | Customer attitudes |
|  | Is there any support, information or training that you would like on ‘*Eat Well @ IGA*’?  *(Free text entry)* | Resources: Staff training ^a^ |
|  | Do you think there will be any problems with maintaining ‘*Eat Well @ IGA*’ at the store in the future?   1. No 2. Yes- please describe | Staff capacity to maintain intervention ^a^ |
|  | Given your knowledge of your store and customers, which of these healthy shopping ideas do you think would be most effective in your store?  Please rank from most (1) to least (7) effective. *(please circle one number per question)*   1. One checkout that doesn’t display unhealthy food 2. All checkouts don’t display unhealthy food 3. Healthier products on display at the ends of aisles 4. Healthy recipes 5. Price discounts on healthy foods and drinks 6. More shelf space for healthy foods and drinks 7. More of catalogue space for healthy foods and drinks | From customer survey  Staff attitudes |
|  | Do you agree that IGA should continue its efforts to encourage healthy eating?  Strongly disagree 1--2--3--4--5--6--7 Strongly agree | From customer survey  Staff attitudes  Continued use ^a^ |
|  | Do you have any other feedback regarding ‘*Eat Well @ IGA*’ (positive or negative, how we could improve etc.)?  *(free text)* | From customer survey  Staff attitudes Unexpected pathways/ consequences |
|  | Are you: *(Circle)* Male / Female / Other | From customer survey  Demographics |
|  | Store: *(tick a box)*  [list of surveyed stores] |  |

# ^a^ Process evaluation constructs identified by: Baranowski, T., & Stables, G. (2000). Process evaluations of the 5-a-day projects. Health Education & Behavior, 27, 157-166
