# Supplementary material for: The ‘Eat Well @ IGA’ healthy supermarket randomised controlled trial: process evaluation
Source: Int J Behav Nutr Phys Act. 2021 Mar 12;18:36. doi: 10.1186/s12966-021-01104-z (PMC7953771; doi:10.1186/s12966-021-01104-z)
Supplement: Supplementary file 3 — Additional file 3. Stakeholder interview discussion guide. Stakeholder interview discussion guide and target construct(s) per question. [file 12966_2021_1104_MOESM3_ESM.docx]

**Additional file 3: Stakeholder interview discussion guide**

**Interviewing technique:**

1. Semi-structured. Questions may be omitted where the content is covered in an answer to another question.
2. Questions below are to be adapted as appropriate for interviewee- including use of language and level of detail. Questions will be deleted as relevant to position.
3. Question allocation: SM- Store manager; MM- Marketing manager; DM, department managers; R, research staff; C, local government council staff; CEO

| **Question** | **Construct/ Purpose ^a^** | **People** |
| --- | --- | --- |
| Can you tell me about your role at [your organisation], and how that relates to the *Eat Well @ IGA* project? | Context/ Partnerships | All |
| Do you think supermarkets influence what their customers eat? | Context | All |
| Can you tell me about your experience with *Eat Well @ IGA*, from when the idea first came up until now?   - What was/is your understanding of the aim of *Eat Well @ IGA*? - If a new staff member joined IGA, how would you explain what *Eat Well @ IGA* is and its purpose? (for floor/ service staff) - Before the store changes started happening, did you personally think that *Eat Well @ IGA* was a good idea? | Context  Motivations: Relative advantage, Compatibility ^a^ | All |
| Can you give me an overview of the different components of *Eat Well @ IGA*?   - How were these developed? | Practicalities of implementation | R |
| Can you tell me about some of the other people or organisations involved in making these changes and what their role was? | Partnerships | All |
| Did you take part in staff training about *Eat Well @ IGA*? Was this helpful? | Intervention components: Staff training | DM |
| Which components did you think were most effective in helping customers choose healthier options? (Trolley and basket signs, Floor signs, Health Star Rating shelf tags, Posters, Shelf signs, letterbox flyers, social media).  Were there any components that were not effective in helping customers choose healthier options or you think could be improved?   - E.g. signage development, messaging, quality of signs/posters etc. | Intervention components: Signage  Adaptations to initial plans ^a^ | SM, MM, DM |
| What do you think was helpful in making sure that *Eat Well @ IGA* happened, and keeping it going in the store?  What do you think was made *Eat Well @ IGA* harder to put in place, and harder to keep it going in the store?  Would any other support from IGA or other organisations have made implementing *Eat Well @ IGA* easier? | Barriers and enablers | MM, CEO, C |
| What were the reactions from customers?   - Have you received any direct feedback? - Did you receive positive feedback? - Did you receive any complaints? - (if no direct feedback) What customer reactions have you observed? - Which components of *Eat Well @ IGA* have prompted the biggest reactions? | Customer perceptions | SM, DM |
| From the time when the first healthy changes were made to the store, have you seen any changes in the types or amount of foods and drinks customers purchase?   - Did the outcomes match your expectations?   Have you noticed any differences in reactions from different groups of customers?   - Younger/ older - Health-conscious customers | Relative sales of healthy versus unhealthy food  Community stewardship  Reach/ socioeconomic differences in response ^b^ | SM, DM, CEO, MM  SM, DM |
| What have the benefits of the project been for IGA?   - Competitive advantage | Competitive advantage **^a^** | SM, CEO, MM |
| Have there been any downsides to participating in *Eat Well @ IGA* for the store/group? | Unintended consequences **^a^** | SM, CEO, MM |
| What were the reactions of staff to *Eat Well @ IGA*?   - Floor staff and checkout staff? - Store managers? | Staff attitudes | DM, SM, MM |
| What do you think the effect has been on the store as a business?   - What if profit had gone down? | Profitability **^a^**  Competitiveness **^a^**  Return on investment **^a^** | SM, CEO, MM |
| Have you discussed *Eat Well @ IGA* with your suppliers?   - What has been their reaction? - Reps versus supplier management? | Attitudes of business stakeholders | MM |
| Have you received feedback from organisations outside IGA?   - [Buyer’s group] - Other IGAs - Other supermarket chains - Media - [Local government] - [Funding body] | Recognition from community and external organisations | CEO, MM, R, C |
| If you were to give advice to another supermarkets interested in influencing customers to make healthier purchases- what would that be?   - Would you advise them to do anything differently? - What if they were setting up a supermarket from scratch?   - Changes to shelf space or store layout? | Adaptations to initial plans **^a^**  Facilitators and enablers | SM, MM, CEO |
| On balance, considering all the benefits and challenges you have described today, do you think that IGA should continue with the program? | Time and/or cost associated with implementation and maintenance | All |
| We are interviewing a few different people for this project. If you were to look at it from the perspective of [council/IGA/other] what do you think they would see as the pros and cons of *Eat Well @ IGA*?   - Can you talk about how your partnership with IGA/ the council/[University] came about and the process of working with them throughout this project? - What were the advantages? - Were there any difficulties? (e.g. lack of understanding of business decisions) | Partnerships | MM, C, CEO |
| Can you describe how this project was funded?  Do you think the store changes could have gone ahead without funding?  What ongoing costs do you foresee for this project? | Partnerships  Costs/ Funding **^a^** | MM, C, CEO |
| What would do you think will happen next for *Eat Well @ IGA*?   - Will the store continue to maintain the changes? Why/ why not? - If yes, do you think there will be any changes to the current initiative components? - Do you think other IGA stores will implement *Eat Well @ IGA*? (senior people)   - Why/ why not?   - If yes, what would be the process for this? | Sustainability  Retailer satisfaction with (support for) initiative  Capacity building  Re-invention **^a^**  Diffusion **^a^** | All  SM, MM, CEO, C, R  “”  “” |
| Based on your experiences in supermarkets, are there any other strategies you think would be feasible and effective in getting people to make healthier food and drink choices in supermarkets? | Re-invention **^a^** | SM, MM, CEO, DM |
| Are there any other comments you’d like to make about *Eat Well @ IGA*? If anything else occurs to you later, you can follow up with me in person or by email. |  | All |

# ^a^ Derived from Diffusion of Innovations theory concept from: Rogers, E.M. (2003). Diffusion of innovations. New York, NY: Free Press.

^b^ Derived from RE-AIM process evaluation framework concept from: Glasgow, R.E., Vogt, T.M., & Boles, S.M. (1999). Evaluating the public health impact of health promotion interventions: the RE-AIM framework. *Am J Pub Health*, 89, 1322-1327.
