# Supplementary material for: The ‘Eat Well @ IGA’ healthy supermarket randomised controlled trial: process evaluation
Source: Int J Behav Nutr Phys Act. 2021 Mar 12;18:36. doi: 10.1186/s12966-021-01104-z (PMC7953771; doi:10.1186/s12966-021-01104-z)
Supplement: Supplementary file 4 — Additional file 4. Stakeholder roles and lines of communication. Figure showing lines of communications between different stakeholders, their places within organisations and main organisational roles. [file 12966_2021_1104_MOESM4_ESM.docx]

**Additional file 4: Stakeholder roles and lines of communication**

**National branding and buyer’s group (wholesale distribution and marketing)**

**Local government (store-researcher-community liaison)**

Research and Evaluation Officer*

**IGA (national brand of Independent Grocers of Australia)**

**Local buyer’s group (recruitment of participating stores)**

**Regional IGA chain (implementation and maintenance of in-store changes)**

Marketing manager*

CEO*

Independent store owners**

Floor staff, service staff

**University (evaluation)**

Research lead*

Store monitors

Research assistants**

Process evaluation lead

Chief Investigator Team

**State government funding body (funding)**

Chain store managers****

Department managers ****

Store managers***

Customers

Data, media and administration support

Advisory Team

**Local community (customers)**

**Federal government funding body (funding)**

Notes: Arrows represent main lines of communication. Key positions specified. Main role of each stakeholder organisation specified in brackets. * indicates an interviewed stakeholder in this role.
