# Supplementary material for: The ‘Eat Well @ IGA’ healthy supermarket randomised controlled trial: process evaluation
Source: Int J Behav Nutr Phys Act. 2021 Mar 12;18:36. doi: 10.1186/s12966-021-01104-z (PMC7953771; doi:10.1186/s12966-021-01104-z)
Supplement: Supplementary file 5 — Additional file 5. Customer survey detailed results. Detailed customer survey results including tabulated overall and subgroup analyses and text descriptions. [file 12966_2021_1104_MOESM5_ESM.docx]

**Additional file 5: Detailed customer survey results**

**Table S1: Between store variation of recall of *Eat Well @ IGA* project components (n=500), October 2017**

| **Project component** | **Intra-cluster correlation coefficient estimate [95%CI]** |
| --- | --- |
| ***Eat Well @ IGA* overall** | 0.067 [0.015, 0.247] |
| **Trolley and/or basket signs** | 0.066 [0.015, 0.240] |
| **Health Star Rating shelf tag** | 0.031 [0.006, 0.157] |
| **Posters** | 0.080 [0.020, 0.268] |
| **Shelf signs** | 0.016 [0.002, 0.144] |
| **Letterbox flyers** | 0.009 [<0.001, 0.522] |
| **Staff t-shirts** | 0.113 [0.029, 0.357] |
| **Social media** | 0.059 [0.009, 0.313] |

IGA, Independent Grocers of Australia.

**Table S2: Customer perceptions of *Eat Well @ IGA* project and components (n=500), October 2017**

| **Project component perception (estimated percentage [95%CI])** | **Overall ^a^ (n=500)** | **Customer demographic subgroups ^b^** | | | | | | | | | |
| --- | --- | --- | --- | --- | --- | --- | --- | --- | --- | --- | --- |
|  |  | **Age** | | **Gender** | | **SEIFA** | | **Education** | | **Store shopping frequency** | |
|  |  | 18-54y (n=172) | 55y and older (n=324) | Male (n=137) | Female (n=357) | Low (n=213) | High (n=285) | High school or lower (n=327) | University or higher (n=165) | Regular (n=136) | Infrequent (n=235) |
| ***Eat Well @ IGA* overall** | 85[81,89] | **80 [72,85]** | **88 [83,92]** | **73 [63,81]** | **90 [86,93]** | **80 [73,86]** | **88 [83,92]** | 834 [78,88] | 88 [81,93] | **86 [81,91]** | **76 [67,84]** |
| **Trolley and/or basket signs** | 75 [69,73] | **69 [60,77]** | **79 [72,84]** | **64 [52,74]** | **80 [73,86]** | **68 [59,76]** | **80 [72,85]** | 73 [66,80] | 78 [69,85] | **76 [68,82]** | **62 [51,71]** |
| **Floor signs** | 71 [61,79] | **62 [50,73]** | **77 [67,84]** | **61 [46,73]** | **75 [65,83]** | 63 [49,75] | 77 [66,85] | 70 [59,79] | 73 [61,83] | 68 [59,75] | 56 [45,66] |
| **HSR shelf-tag** | 80 [73,85] | **73 [63,81]** | **84 [78,90]** | **69 [57,78]** | **85 [78,89]** | **72 [64,79]** | **85 [80,89]** | 79 [73,85] | 81 [72,88] | **81 [72,87]** | **67 [54,78]** |
| **Posters** | 77 [72,81] | 71 [63,78] | 80 [74,85] | **68 [58,77]** | **81 [75,85]** | **71 [63,78]** | **80 [74,85]** | 78 [72,83] | 74 [65,81] | **82 [75,88]** | **61 [51,71]** |
| **Shelf signs** | 77 [72,82] | **71 [62,78]** | **82 [76,87]** | **66 [545,75]** | **82 [76,87]** | **70 [62,78]** | **82 [76,86]** | 78 [71,83] | 78 [69,84] | **81 [73,87]** | **61 [50,72]** |
| **Letter box flyers** | 58 [48,68] | **46 [34,58]** | **66 [55,75]** | **48 [35,62]** | **62 [52,72]** | 49 [35,63] | 65 [53,76] | 61 [50,71] | 53 [40,66] | **60 [51,68]** | **35 [25,48]** |
| **Social media** | 55 [45,64] | **47 [36,59]** | **60 [49,70]** | **38 [25,52]** | **62 [52,72]** | **46 [35,57]** | **61 [51,70]** | 58 [47,68] | 49 [37,62] | 54 [44,63] | 44 [32,56] |

All questions scored on Likert scale from 1 (Strongly dislike) to 7 (Strongly like) and dichotomised into Like (5 to 7) compared to Do not like (1 to 4) for analysis. Bolding indicates significant difference (p <0.05) between subgroups. HSR, Health Star Rating; IGA, Independent Grocers of Australia; SEIFA, Index of Relative Socio-Economic Disadvantage- Socio-Economic Indexes for Areas. ^a^ Estimated using univariate logistic models with store as random effect to account for the clustering induced by store. ^b^ Estimated using univariate mixed-effects logistic models with the demographic variable as a fixed effect and store as random effect to account for the clustering induced by store.

**Table S3: Customer support for future interventions and effect on customer loyalty (n=500), October 2017**

| **Agreement (estimated percentage [95%CI])** | **Overall ^a^ (n=500)** |  | **Customer demographic subgroups ^b^** | | | | | | | | | | |  |
| --- | --- | --- | --- | --- | --- | --- | --- | --- | --- | --- | --- | --- | --- | --- |
|  |  | **Age** | | **Gender** | | **SEIFA** | | **Education** | | **Store shopping frequency** | | | |  |
|  |  | 18-54y (n=172) | 55y and older (n=324) | Male (n=137) | Female (n=357) | Low (n=213) | High (n=285) | High school or lower (n=327) | University or higher (n=165) | Regular (n=136) | Infrequent (n=235) | | |  |
| **Support for proposed changes** | | | | | | | | | | | | | |  |
| One checkout without unhealthy food ^c^ | 68 [61,74] | 70 [61,78] | 66 [59,73] | **57 [47,67]** | **72 [65,78]** | 63 [55,71] | 71 [64,77] | **64 [56,71]** | **75 [66,82]** | 66 [58,72] | 68 [58,76] | |  |  |
| All checkouts without unhealthy food ^c^ | 61 [56,65] | 58 [51,65] | 63 [57,68] | **49 [40,57]** | **65 [60,70]** | 58 [52,65] | 63 [57,68] | 57 [52,63] | 67 [59,74] | 59 [53,65] | 57 [48,65] | |  |  |
| Healthier products at the ends of aisles ^c^ | 82 [79,86] | 86 [80,90] | 81 [76,85] | **75 [67,82]** | **86 [82,89]** | 82 [76,87] | 83 [78,87] | 80 [75,84] | 87 [80,91] | 81 [75,86] | 82 [75,88] | |  |  |
| Healthy recipes ^c^ | 88 [84,91] | 91 [85,94] | 87 [82,90] | **82 [75,88]** | **90 [86,93]** | 89 [84,92] | 87 [82,90] | 86 [81,90] | 92 [86,95] | 88 [83,92] | 88 [81,92] | |  |  |
| IGA should continue to encourage healthy eating ^c^ | 97 [93,99] | 96 [90,98] | 98 [94,99] | 97 [91,99] | 97 [93,99] | **94 [90,96]** | **98 [96,99]** | **96 [91,98]** | **100 [96,100]** | 96 [92,98] | 97 [92,99] | |  |  |
| **Customer loyalty effects** | |  |  |  |  |  |  |  |  |  |  |  | | |
| More likely to shop at IGA due to *Eat Well @ IGA* ^c^ | 55 [45,65] | 57 [45,68] | 54 [44,65] | **47 [35,60]** | **58 [47,68]** | **46 [36,57]** | **61 [52,69]** | 55 [44,65] | 55 [43,66] | **61 [52,69]** | **46 [36,56]** | |  |  |
| Positive or neutral feedback on *Eat Well @ IGA* ^d^ | 98 [82,100] | 99 [81,100] | 98 [80,100] | 99 [78,100] | 98 [80,100] | 97 [62,100] | 98 [86,100] | 100 [88,100] | 97 [70,100] | 99 [84,100] | 93 [61,99] | |  |  |
| Increased store shopping frequency ^e^ | 7 [5,9] | **10 [6,15]** | **5 [3,8]** | 6 [3,11] | 7 [5,10] | 5 [3,10] | 6 [3,9] | 7 [5,11] | 5 [3,10] | 8 [6,12] | 4 [2,8] | |  |  |
| New customer because of *Eat Well @ IGA* ^f^ | 3 [1,6] | 4 [1,10] | 3 [1,6] | 5 [2,12] | 2 [1,6] | 5 [2,15] | 1 [0.3,6] | 3 [1,7] | 2 [1,7] | 3 [1,6] | 3 [1,8] | |  |  |

^a^ Estimated using univariate logistic models with store as random effect to account for the clustering induced by store. ^b^ Estimated using univariate mixed-effects logistic models with the demographic variable as a fixed effect and store as random effect to account for the clustering induced by store. **^c^** Scored on Likert scale from 1 (Strongly disagree) to 7 (Strongly disagree), and dichotomised into Support (5 to 7) compared to Do not support (1 to 4) for analysis. ^d^ Free-text responses coded into positive or neutral compared to negative. ^e^ Responses dichotomised into Increased Frequency (More often) compared to Not Increased (Less often /No change) for analysis. ^f^ Responses dichotomised into Yes compared to No/Unsure. Bolding indicates significant difference (p <0.05) between subgroups. HSR, Health Star Rating; IGA, Independent Grocers of Australia; SEIFA, Index of Relative Socio-Economic Disadvantage- Socio-Economic Indexes for Areas.

**Detailed customer survey results description**

Recall and influence

Overall respondent and sub-group recall of project components is found in **Table 2**. Compared to those aged 18 to 54 years, those aged 55 years and older were less likely to recall Health Star Rating (HSR) shelf tags (p=0.03), posters (p<0.01), and shelf signs (p<0.01). There were no age differences in recall of the overall project, social media, t-shirts, flyers, or trolley or basket signs.

Compared to infrequent shoppers, regular shoppers were up to twice as likely to recall the project overall (p=0.05), trolley and basket signs (p=0.03), shelf signs (p=0.05) and letter box flyers (p<0.01). Differences were not significant for HSR shelf tags, posters, social media or staff t-shirt recall.

There was no difference in project or component recall between gender, educational attainment or Socio-economic Index for Areas (SEIFA) levels.

Perception of *Eat Well @ IGA* components

Overall respondent and sub-group perceptions of project components are summarised in **Table S2**. Older respondents generally rated study components more highly than younger respondents. Compared to respondents 18 to 54 years old, respondents 55 years and older rated the project overall and all components except posters more highly (all p≤0.06).

Female respondents rated the project overall and all components more highly than males (all p<0.04). Regular IGA shoppers rated all components more highly than infrequent shoppers (all p≤0.03), except floor signs (p=0.09) and social media.

Participants with a higher SEIFA level rated the project as a whole (p=0.03) and all components more highly than those with a lower SEIFA level (all p≤0.06).

There was no difference in perception of project components by educational status.

Suggestion for further healthy eating initiatives

Overall respondent and sub-group suggestions for further healthy eating initiatives are summarised in **Table S3**. Those who completed a university degree or higher were more supportive of one checkout without unhealthy food at IGA (p=0.02), as were female shoppers (p<0.01). Female shoppers were also more supportive of all checkouts without unhealthy food and healthier products at the end of aisles (both p<0.01), and healthy recipes (p=0.02). No other subgroups differences were seen for support of additional healthy eating initiatives. However, all subgroups had an estimated percentage of at least 75% support for healthier products at the end of aisles and inclusion of healthy recipes. All subgroups had at least 94% support for IGA continuing its efforts to encourage healthy eating.

Regular IGA shoppers were more likely to say that *Eat Well @ IGA* increased their likelihood of shopping at IGA (p=0.01), as were female customers compared to male customers (p=0.05), and high compared to low SEIFA customers (p=0.02).

Effect on customer loyalty

Ten percent [95%CI:6,15] of customers aged 18 to 54 years old reported that they had increased shopping frequency at IGA due to the project, compared to 5% [95%CI:3,8] customers 55 years and older (p=0.03). There were no differences found between any subgroups (age, gender, education level, SEIFA, shopping frequency) on whether the respondent was a new customer as a result of the project.
