# Supplementary material for: The ‘Eat Well @ IGA’ healthy supermarket randomised controlled trial: process evaluation
Source: Int J Behav Nutr Phys Act. 2021 Mar 12;18:36. doi: 10.1186/s12966-021-01104-z (PMC7953771; doi:10.1186/s12966-021-01104-z)
Supplement: Supplementary file 6 — Additional file 6. Staff survey detailed results. Detailed staff survey results including tabulated overall and subgroup analyses. [file 12966_2021_1104_MOESM6_ESM.docx]

**Additional file 6: Staff survey detailed results**

**Table S4: Staff survey participant demographic and employment characteristics (n=82 respondents)**

| **Characteristic** | **n (%) respondents** | | |
| --- | --- | --- | --- |
|  | **Overall (n=82)** | **Intervention stores (n=40)** | **Control stores (n=42)** |
| **Age** |  |  |  |
| <25 years | 32 (39) | 18 (45) | 14 (33) |
| 25-44 years | 21 (26) | 8 (20) | 13 (31) |
| 45- 64 years | 22 (27) | 10 (25) | 12 (29) |
| 65+ years | 1 (1) | 0 (0) | 1 (2) |
| Missing | 6 (7) | 4 (10) | 2 (5) |
| **Gender** |  |  |  |
| Male | 18 (22) | 13 (33) | 5 (12) |
| Female | 51 (62) | 21 (53) | 30 (71) |
| Other | 6 (7) | 3 (8) | 3 (7) |
| Missing | 7 (9) | 3 (8) | 4 (10) |
| **Role** |  |  |  |
| Store manager | 4 (5) | 2 (5) | 2 (5) |
| Department manager | 23 (28) | 13 (33) | 10 (24) |
| Floor staff | 15 (18) | 7 (18) | 8 (19) |
| Deli staff | 5 (6) | 3 (8) | 2 (5) |
| Checkout | 15 (18) | 6 (15) | 9 (21) |
| Administration | 3 (4) | 1 (2) | 2 (5) |
| Other | 4 (5) | 2 (5) | 2 (5) |
| Multiple roles | 9 (11) | 5 (13) | 4 (10) |
| Missing | 4 (5) | 1 (2) | 3 (7) |
| **Length of time employed at IGA** | | | |
| Less than 6 months | 4 (5) | 2 (5) | 2 (5) |
| 6 months to 1 year | 4 (5) | 1 (2) | 3 (7) |
| 1 to 2 years | 8 (10) | 5 (13) | 3 (7) |
| 2 years or more | 65 (79) | 31 (78) | 34 (81) |
| Missing | 1 (1) | 1 (2) | 0 (0) |
| **Employment fraction** |  |  |  |
| Full-time | 35 (43) | 19 (48) | 16 (38) |
| Part-time | 14 (17) | 2 (5) | 12 (29) |
| Casual | 30 (36) | 17 (43) | 13 (31) |
| Missing | 3 (4) | 2 (5) | 1 (2) |
| **Main shop at IGA** |  |  |  |
| Always | 21 (26) | 6 (15) | 15 (36) |
| Usually | 20 (24) | 7 (18) | 13 (31) |
| Sometimes | 22 (27) | 15 (38) | 7 (17) |
| Rarely or never | 15 (18) | 8 (20) | 7 (17) |
| N/A (not main shopper) | 2 (2) | 2 (5) | 0 (0) |
| Missing | 2 (2) | 2 (5) | 0 (0) |
| **Involved with *Eat Well @ IGA*** |  |  |  |
| Yes | 13 (16) | 8 (20) | 5 (12) |
| No | 67 (82) | 31 (78) | 36 (86) |
| Missing | 2 (2) | 1 (3) | 1 (2) |

**Table S5. Staff overall perceptions of *Eat Well @ IGA* project components**

| **Component** | **Median [inter-quartile range] perception** | | |
| --- | --- | --- | --- |
|  | **Overall (n=82)** | **Intervention stores (n=40)** | **Control stores (n=42)** |
| *Eat Well @ IGA* overall | 6 [5,7] | 6 [5,7] | 6 [5,7] |
| Trolley and basket signs | 6 [5,7] | 6 [4,6] | 7 [5,7] |
| Floor signs | 5 [4,7] | 5 [4,6] | 6 [4,7] |
| Health Star Rating shelf tags | 5 [4,7] | 5 [3,6] | 5 [4,7] |
| Posters | 6 [4,7] | 5 [4,6] | 6 [5,7] |
| Shelf signs | 5 [4,6] | 5 [4,6] | 5 [4,6] |
| Flyers | 5 [2,6] | 5 [3,5] | 5 [4,7] |
| Aisle fins | 4 [3,6] | 5 [3,6] | 5 [4,7] |
| Social media | 5 [4,6] | 5 [4,6] | 6 [4,7] |

Questions on Likert scale from 1 (strongly dislike) to 7 (strongly like)

**Table S6. Staff free-text reporting of components that worked particularly well**

| **Component** | **n (%) respondents** | | |
| --- | --- | --- | --- |
|  | **Overall (n=82)** | **Intervention stores (n=40)** | **Control stores (n=42)** |
| Trolley/ basket signs | 22 (27) | 11 (28) | 11 (26) |
| Health Star Rating shelf tags | 9 (11) | 5 (13) | 4 (10) |
| Floor signs | 9 (11) | 6 (15) | 3 (7) |
| Posters | 3 (4) | 1 (3) | 2 (5) |
| Shelf signs | 1 (1) | 1 (3) | 0 (0) |
| Social media | 1 (1) | 1 (3) | 0 (0) |
| Staff t-shirts | 1 (1) | 1 (3) | 0 (0) |
| Aisle fins | 0 (0) | 0 (0) | 0 (0) |
| Non-specific components | 6 (7) | 0 (0) | 6 (14) |

Note: staff could list multiple components.

**Table S7. Staff free-text reporting of components that did not work well**

| **Component** | **n (%) respondents** | | |
| --- | --- | --- | --- |
|  | **Overall (n=82)** | **Intervention stores (n=40)** | **Control stores (n=42)** |
| Health Star Rating shelf tags | 14 (17) | 7 (18) | 7 (17) |
| Shelf signs | 8 (10) | 5 (13) | 3 (7) |
| Flyers | 5 (6) | 2 (5) | 2 (5) |
| Trolley/ basket signs | 3 (4) | 3 (8) | 0 (0) |
| Floor signs | 3 (4) | 1 (3) | 2 (5) |
| Social media | 2 (2) | 1 (3) | 1 (2) |
| Aisle fins | 0 (0) | 0 (0) | 0 (0) |
| Posters | 2 (2) | 1 (3) | 1 (2) |
| Staff t-shirts | 1 (1) | 1 (3) | 0 (0) |
| Non-specific components | 6 (7) | 2 (5) | 4 (10) |

Note: staff could list multiple components.

**Table S8. Customer feedback to staff about *Eat Well @ IGA* or its components**

| **Frequency of customer feedback** | **n (%) respondents** | | |
| --- | --- | --- | --- |
|  | **Overall (n=82)** | **Intervention stores (n=40)** | **Control stores (n=42)** |
| Often | 1 (1) | 1 (3) | 0 (0) |
| Sometimes | 13 (16) | 10 (25) | 3 (8) |
| Rarely | 20 (20) | 11 (28) | 9 (23) |
| Never | 46 (58) | 18 (45) | 28 (70) |
| **Customer feedback positivity to staff who received feedback** | | | |
| Very positive | 9 (12) | 7 (18) | 2 (5) |
| Slightly positive | 17 (22) | 13 (33) | 4 (10) |
| Neutral | 12 (15) | 5 (13) | 7 (18) |
| Slightly negative | 1 (1) | 0 (0) | 1 (3) |
| Very negative | 0 (0) | 0 (0) | 0 (0) |
| No feedback received | 39 (50) | 14 (36) | 25 (64) |

**Table S9. Staff respondent rankings of perceived effectiveness of further healthy changes in encouraging customer healthy choices**

| **Component** | **Median [inter-quartile range] perception** | | |
| --- | --- | --- | --- |
|  | **Overall (n=54)** | **Intervention stores (n=27)** | **Control stores (n=27)** |
| One checkout that doesn’t display unhealthy food | 7 [6,7] | 7 [6,7] | 6 [4,7] |
| All checkouts don’t display unhealthy food | 6 [3,6] | 6 [2,6] | 6 [4,7] |
| Healthier products on display at the ends of aisles | 3 [2,5] | 3 [2,5] | 4 [2,6] |
| Healthy recipes | 4 [2,5] | 4 [2,5] | 3 [2,5] |
| Price discounts on healthy foods and drinks | 2 [1,3] | 2 [1,3] | 2 [1,3] |
| More shelf space for healthy foods and drinks | 4 [4,5] | 4 [4,5] | 4 [4,5] |
| More of catalogue space for healthy foods and drinks | 3 [3,5] | 3 [3,5] | 4 [2,5] |

Ranked from 1 (most effective) to 7 (least effective)
