# Supplementary material for: The ‘Eat Well @ IGA’ healthy supermarket randomised controlled trial: process evaluation
Source: Int J Behav Nutr Phys Act. 2021 Mar 12;18:36. doi: 10.1186/s12966-021-01104-z (PMC7953771; doi:10.1186/s12966-021-01104-z)
Supplement: Supplementary file 7 — Additional file 7. Stakeholder interview themes, sub-themes and illustrative quotes. Stakeholder interview themes, sub-themes and illustrative quotes. [file 12966_2021_1104_MOESM7_ESM.docx]

**Additional file 7: Stakeholder interview themes, sub-themes and illustrative quotes**

| **Sub-theme** | **Sub-theme** | **Illustrative quote** |
| --- | --- | --- |
| **Overarching theme 1: CONSIDER THE INFLUENCE OF INDUSTRY AND PUBLIC HEALTH CONTEXT ON INTERVENTION** | | |
| Supermarket operations | Status quo is unhealthy | “those unhealthy foods are … promoted more often than any other product in our store and that's just the way it is.” (IGA Executive)  “with the aspects of the projects that we've been able to implement, I don't think some of them are strong enough to overcome the marketing that is happening in store.” (Researcher) |
|  | Power of conglomerates | “How do you change the world? Because you're changing the world, yeah, how do you change it when these people are big conglomerates, big companies, and they do the market research. They're not interested in health, they're interested in selling another chocolate bar.” (IGA Executive) |
|  | Supplier contracts | “We have a little bit of freedom here, but most of what's up the [front-of-aisle display] is trading terms with those suppliers, so we have to comply.” (Store Manager) |
|  | Store variability | “But all stores are all different shapes and sizes. In the bigger shops, it probably didn't look as cluttered. Whereas in the smaller shop like mine, it just looks too busy when you see all those tickets and posters.” (Store Manager) |
| Industry | Other supermarkets | “Then we had the competition open up in October. And we've probably been flat. That took a bit of our cream off the top” (Store Manager) |
|  | Customer trends | “I think the whole market's starting to change… people gravitating towards all those healthier type products. So I think within a couple of years, the demand for those products – well, it's already there now, but it's going to be even stronger.” (Store Manager) |
|  | Industry change | “I think it's a good thing, yeah I think it's – you know like Coles and Woolies [major supermarket competitors] sort of do it, you know, the healthy eating sort of thing. I think we should be supporting healthy eating for sure.” (Store Manager). |
|  | Business model | “The other thing that I've learned through this has been although they're committed, they're not so committed that they're willing to change the whole business model” (Local Government Research Officer) |
| Influences on customer choice | Price | “it's got to the stage where customers, they won't buy it unless it's on special because they've been trained.” (IGA Executive) |
|  | Health knowledge and interest | “I think they aren’t that concerned about it, they probably just come and buy what they want to buy and they’re not that worried about a health star rating or a program that the store is running, they just want to get in and do their shopping and go home.” (Store Manager) |
|  | Customer awareness of initiative | “How many people knew about it and actually really knew about it? Very few. Now we've had those decals on the floor for how long?” (IGA Executive) |
|  | Kids | “…shoppers are my age and that have young kids that are trying to get their kids to eat better and that sort of thing.” (Store Manager) |
|  | Consumer trends | “People's eating habits are changing. The fresh food mix of the supermarket is increasing dramatically” (Store Manager) |
| Public health advocacy | Responding to community health needs | “…our demographics around here, it's probably - if you just make people make one choice differently, it's a winner that way.” (Store Manager) |
|  | Supporting organisations and programs | “Bendigo was one of 12 or 14 sites that was given Healthy Together funding. So we were called Healthy Together Bendigo but we sat under the broader Healthy Together Victoria initiative, and that was a huge investment in population health and prevention” (Local Government Research Officer) |
| **Overarching theme 2: DEVELOP PROJECT PARTNERSHIP AND ALIGN STAKEHOLDER OBJECTIVES** | | |
| Stakeholders | Deakin University | “if we had to rely on our resources to hang the [point-of-sale material], put the trolley signs up, hang the tickets, identify the products, maintaining that, it wouldn’t have worked. Without the support and the ongoing support from Deakin it wouldn’t have maintained moving forward.” (IGA Executive) |
|  | Local government research officer | “[the Local Government Research Officer]’s been a key person in terms of the partnerships and the relationships with the IGA supermarkets, being that person who is local and really understands what's happening around Bendigo and how it's actually going in the store, and she's also really good in terms of the project itself and what's happening and how it can be expanded, and the next steps and things that can take place.” (Researcher) |
|  | Marketing manager | “being the merchandise manager for the group it was very much a case of getting my thoughts and how- because it was directly involved in category- and how potentially the store would be merchandised it’s sort of direct involvement with me.” (IGA Executive) |
|  | Store managers | “…as the store manager. My role is to support [Deakin] in implementing the program which was come up with Deakin and in conjunction with our head office, after they'd rolled it out in other stores. So my purpose was to oversee it being implemented and maintained during the time here.” (Store Manager) |
|  | IGA network | “they were already connected through a collective buying group…, so they had those really close relationships with the other stores that we now work with” (Local Government Research Officer) |
| Expertise | Research expertise | “it does require really rigorous evaluation and really rigorous data analysis and those are just skills we don't have in local government. More than that, the people on the ground - it's pretty much just me in local government supporting this at an on-the-ground level. I mean, there's strategic support and that type of thing from above but I couldn't have done this with IGA on my own. We needed that expert support from Deakin around the evidence, the data analysis, ideas about what would work, all those types of things.” (Local Government Research Officer) |
|  | Nutrition expertise | “there can be a lot of nuances with nutrition, which I don't always think other people might see. … I guess like legumes or that type of little thing - other people may not see that as a big deal or something, but from a dietitian perspective you know that that is a really great food to encourage from so many different aspects.” (Researcher) |
|  | Supermarket operations knowledge | “…being the merchandise manager for the group it was very much a case of getting my thoughts and how- because it was directly involved in category- and how potentially the store would be merchandised it’s sort of direct involvement with me.” (IGA Executive) |
| Partnerships | Aligning stakeholder objectives | “One, the whole objective of the campaign was to try and influence or encourage people to eat healthier but also it was a win/win in the means of if we’re selling more of those 4.5, 5-star type products within our stores there’s the potential of … higher profit…” (IGA Executive) |
|  | Collaboration | “the aims of the trial were worked out in collaboration between the three partners at a series of meetings where we came up with - we looked at the research in the area and talked about different options and came up with a series of trials that we thought were feasible.” (Researcher) |
|  | Ownership | “[the executive-driven approach has] also taken a little bit away from that ownership among the stores themselves” (Local Government Research Officer) |
|  | Conflict of interest | “I think it's important to note that we haven't taken any money from [IGA], and that really limits conflict in one way. And I think, because we've got a partnership, which includes not only Bendigo but also VicHealth [the state health promotion agency] and a range of academic investigators, I think that's really helped to make sure that the decisions are based on the public health interest rather than – we're not just serving the IGA interests.” (Researcher) |
| Engagement | Staff engagement | “The staff loved it. They liked the concept of it and they can see the benefits as well, not just for them but for our customer. I mean, and our customer could be their mum, their brother, their sister, do you know what I mean, so it sort of hit home to them.” (Store Manager) |
|  | Stakeholder relationships | “my biggest role is sort of that partnership management, … providing an independent mediating party … I'm often the person who softly, softly makes those suggestions, trying to align them with IGA's perspective and their needs and things like that, and same in the other direction” (Local Government Research Officer) |
|  | Community engagement | “lots of community organisations … literally are thankful and grateful that we were doing the program, and obviously they were going to go back to their organisations and spread the word about IGA doing this and that's fantastic.” (Store Manager) |
|  | IGA executive driven | “it was a very top-down approach where [the former Executive Chairman] said, "Our stores are doing this," and then [the IGA marketing manager] and the store managers had to make it work.” (Local Government Research Officer)  “Yeah and that's why I say always start with us, what do we need to do, because it's easy to blame someone else but what do we need to do to get this program going.” (IGA Executive) |
| Publicity | Launch | “Any publicity is good publicity.... So to be seen to be trying to do the right thing is a good thing at the end of the day.” (Store Manager) |
|  | Credibility | “So having Deakin and Bendigo promoting what they're doing I think has been the key thing. That they realise “Actually, we couldn't do this by ourselves. We've needed that – what's the word – ‘credibility’.” (Researcher) |
| **Overarching theme 3: CO-DESIGN AND COLLABORATE FOR INTERVENTION DESIGN AND PROGRESSION** | | |
| Aims | Win-wins | “I think it was clear that our interests and their interests aligned in that we wanted to promote purchase of healthy food, which is fresh food - not exclusively, but a lot of it is fresh food - and they wanted to sell more fresh food because it encourages purchasing of everything in the store, and also because there's issues with wastage there as well. So pretty clear that our interests aligned in relation to that. IGA also, I think, could see that this was going to be good for their corporate image.” (Researcher) |
|  | Collective initiative to improve community wellbeing | “Yeah. If it's us, we're just after - everyone thinks that we're just here to sell things and make money and retire. So, whatever we suggest, we're probably doing it for our financial advantage, not for their benefit and the fact is, it's quite different to that. We'd like to see more people being healthy.” (Store Manager) |
| Momentum | Passionate individuals | “Also, it’s very much got to do with [the research lead]’s …continued involvement I think has helped towards driving it and continue to drive it. The big thing for me is just maintaining that momentum. I think that’s the biggest challenge for us” (Local Government Research Officer) |
|  | Funding | “if there was a partnership with Deakin still and we hadn't been able to find any funding, I think the City [of Greater Bendigo] would have been willing to try in a scaled down form of what we were doing. As I said, a lot of the initiatives that we're doing aren't very expensive…But those things would have been possible but we would never have got the outcome that we've been able to get, certainly in terms of the analysis and the amount of resources that Deakin's been able to draw in” (Local Government Research Officer) |
|  | Feedback | “Yeah, well all of those things affect the timelines, and so even winning grants. You apply for a grant and then you don't hear for nine months, and so you can't start your project for ages. So all of those things affect the timelines, and so, for instance, we're analysing the data at the moment but just to categorise the data from all those stores is taking us a very long time. So the IGA guys would imagine all you need is the - like, they have categories for their store and so they can just print out a - from the data - print out a bar chart or whatever and look at trends. So to categorise every product again, and separately, based on core and discretionary or whatever it is, how healthy the food is, is hard for them to understand, that that's necessary, and that really does blow out the timelines.” (Researcher) |
| Planning | Selecting intervention components | “So we sort of all sat down and discussed a range of options for what we could try in those stores and together we decided on the health star rating shelf tags, the trolley signs and the - is it the floor signs?” (Local Government Research Officer) |
|  | Staged approach | “we didn't want to scare them by proposing that long-term partnership in the early days” (Local Government Research Officer) “Deakin and the City [of Greater Bendigo] always knew that our intention was to try and scale that up to more stores and to test those successful interventions as a combined package in a larger number of stores” (Local Government Research Officer) |
| **Overarching theme 4:IDENTIFY INTERVENTION COMPONENT CHARACTERISTICS, EFFECTIVENESS AND CHALLENGES** | | |
| Point-of-sale interventions | Trolley and basket signs | “having them on the front of the shopping trolleys and the baskets is really good as well because it's in your face” (Department Manager) |
|  | Health Star Rating shelf tags | “people can actually see it and they go, ‘That is a good choice to go for.’” (Department Manager)  “just with the signage falling down, the tickets falling down because they were constantly falling down, we have to redo them. The good thing was that they were colour coded so that was a good thing, otherwise we would have been struggling to find where it was supposed to be. But yeah just something that was actually better, how do you say, easy to maintain, yeah that was the hardest part of them falling.” (Department Manager) |
|  | Salience of point-of-sale material decreases over time | “people just become blind. Like once they have seen it, they have seen it a few times, unless it has changed or altered, they just don’t see it anymore” (Department Manager) |
|  | Visual noise in the supermarket | “I think sometimes when you see a lot of tickets around the shop and posters all around the departments - we sometimes use the term ‘air pollution’, because sometimes it's just too busy and people don't stop and read. But the baskets and the trollies; it's basically right in the customer’s face.” (Store Manager) |
|  | Congruence | “so you're coming in every week and I've got all these *Eat Well* posters and everything around the shop, but as soon as you walk in, all you can see is Coke on an end, chocolate biscuits on an end, chips and all that type of thing.” (Store Manager) |
|  | Modifications | “they'd basically made their own tape or data strip tape to actually sit inside [the shelf strips], like we did with the eggs and the water, but just a general *Eat Well @ IGA* one. And they suggested to me that if there's going to be an area that have lots of 4.5- and 5-star products, why not make one like that?” (Researcher) |
| New project suggestions | Price promotions on healthy products | “I think that was a good first step. Now I think this second step, we need to start looking at those [healthy] products, putting discounts around those products, so that we can actually give [customers] a better reason to change.” (IGA Executive) |
|  | Catalogue | “It might still just be a catalogue special, but if it was a 4.5 or 5 star rating product, put that in the advertising as well, a little blurb on there about it, the initiative of what you're trying to push and get people to eat healthier.” (Store Manager) |
|  | Advertising | “You can't tell customers what to buy but you can obviously suggest to them through advertising and so forth, so how do people know about the products, how do people know about the program?” (Store Manager) |
| **Overarching theme 5: CONSIDER THE COMMERCIAL AND COMMUNITY OUTCOMES** | | |
| Customer response to initiative | Customer feedback | “Some people quite liked it…I wouldn't say it was <long pause> overwhelming.” (Store Manager) |
|  | Customer purchase effects | “basket sales were higher in the produce. It’s a hard to explain one because it does go up and down but we did notice that a lot of people were cooking a lot more, that was one of the things.” (Store Manager)  “People will still buy a hell of a lot of Coke” (Store Manager) |
| Profit impact | Business risk | “Actually, we were a bit nervous that it might be financially bad for us, that if everyone stopped drinking Coke and stopped taking packets of chips and all that stuff but I don't think that will happen.” (Store Manager) |
|  | Profit margins are higher on healthy products | “One, the whole objective of the campaign was to try and influence or encourage people to eat healthier but also it was a win/win in the means of if we’re selling more of those 4.5, 5 star type products within our stores there’s the potential of getting a gain through higher profit because of the associated lines with that.” (IGA Executive) |
|  | Profit is from unhealthy food | “we've got to put a chocolate because we've got to survive, we've got to make money to keep our staff employed and pay our bills.” (IGA Executive) |
| Return on investment | Cost-benefit | “the question for us as a business is what's it going to cost and then what's the return on it?” (IGA Executive)  “I guess at the end of the day it all comes down to money” (Store Manager) |
|  | Corporate image | “one of the other reasons why we embraced the project is it sort of helps like brand equity is massive for any business and it sort of just helps not empower it but it just helps the wellbeing of a brand.” (IGA Executive) |
|  | Competitive advantage | “[The store managers] could see it as, you know, we’re all looking for a bit of a point of difference within our business, they saw it as that so they embraced it as well.” (IGA Executive) |
|  | Time as a resource | “The biggest one would be about the shelf tag, yeah, that’d be the hardest one to maintain without resources and that’s something that we’d have to allocate, we’d have to justify the investment associated with allocating that type of resource to maintain that’ (IGA Executive) |
|  | Priorities | “as a business, there's other things we'd probably focus on with ticketing and things like that, if we had to put the labour into it and stuff like that.” (Store Manager) |
| **Overarching theme 6: PLAN AND ADAPT FOR THE FUTURE USING EVIDENCE AND SUPPORT** | | |
| Ongoing modification | | “So, I think it's a good first step but you don’t want people to get complacent and think that because they’re doing this they can continue to have junk food at the check-outs or that they continue to heavily promote unhealthy food on the premise that, oh, well, at least we're doing *Eat Well @ IGA*. This should be the first step to opening up the conversation about actually having healthier supermarkets.” (Researcher) |
| Sustainability | | “So, it definitely will still need input from Deakin to make sure - even if it's an emailed list of, ‘These are in. These are out,’ once a month, once a quarter or whatever…Otherwise two years down the track, you probably wouldn't recognise the program you've got out there now.’ (Store Manager)  “Like I said, for the first 12 months when they were doing the in-checks and actually coming in every now and then to fix up the ticketing - if we have support like that, I would agree. If we had to manage the entire system ourselves, probably not. There are other things we'd probably try and focus on.” (Store Manager) |
| Scale-up | | “So I'm hopeful that there'll be some scale-up of the project. I think that's what we want to happen first but there are all these - maybe seven or eight different IGAs or people working with IGAs have contacted us to try and use our ideas, and so that - if it doesn't go up to a state or a national level, that may happen too.” (Researcher)  “Yeah, I think if the results come back are good and everything’s positive, I think the potential’s huge more so from a Macro or IGA brand. We’ll probably start to share that a little bit more from a state and from a national perspective.” (IGA Executive)  “If you applied it across every single store in Australia, I think first, honestly speaking, I don’t think a lot of retailers would go for it, unless it’s funded by someone else.” (Store Owner) |
| Evidence | Knowledge translation | “I think there is a lot of potential for it to be taken up more broadly, and I also think that other supermarkets in Australia and around the world are looking to see what the results say and how they can apply it to what they do as well.” (Local Government Research Officer) |
|  | Uncertainty | “But end of the day, it’s like we have to weigh up whether or not it benefits us or not and if it doesn’t, then there’s no point in implementing it, obviously, so yeah.” (Store Owner) |
